# Macrophage TREM2 is a triglyceride-rich lipoprotein receptor that undergoes shedding in prediabetic hepatic steatosis

**DOI:** 10.1172/jci.insight.202675

**Published:** 2026-08-27

**Authors:** Jingjing Tang, Jenny E. Kanter, Baohai Shao, Masami Shimizu-Albergine, Farah Kramer, Ah Reum Khang, Jason Luo, Huaqing Zheng, Alan Tran, Jocelyn Cervantes, Jeremy M. Frey, Mauricio D. Dorfman, Cheng-Chieh Hsu, Laura J. den Hartigh, Tomas Vaisar, Brandon S.J. Davies, Adam E. Mullick, George N. Ioannou, Gordon I. Smith, Samuel Klein, Nicholas O. Davidson, Karin E. Bornfeldt

**Affiliations:** 1 Department of Medicine, Division of Metabolism, Endocrinology and Nutrition, University of Washington Medicine Diabetes Institute, University of Washington, Seattle, Washington, USA.; 2 Department of Biochemistry and Molecular Biology, and; 3 Fraternal Order of Eagles Diabetes Research Center, University of Iowa, Iowa City, Iowa, USA.; 4 Ionis Pharmaceuticals, Carlsbad, California, USA.; 5 Research and Development and Division of Gastroenterology, Veterans Affairs Puget Sound Health Care System, Seattle, Washington, USA.; 6 Division of Gastroenterology, University of Washington, Seattle, Washington, USA.; 7 Department of Medicine, Division of Nutritional Science & Obesity Medicine, and; 8 Division of Gastroenterology, Washington University School of Medicine, Saint Louis, Missouri, USA.; 9 Department of Laboratory Medicine and Pathology, University of Washington Medicine Diabetes Institute, University of Washington, Seattle, Washington, USA.

**Keywords:** Metabolism, Vascular biology, Atherosclerosis, Lipoproteins, Macrophages

## Abstract

Prediabetes is associated with increased production of triglyceride-rich lipoproteins (TRLs), cardiovascular disease (CVD), and hepatic steatosis, which is linked to increased plasma levels of soluble TREM2 (sTREM2), the shed domain of TREM2 (triggering receptor expressed on myeloid cells 2). Whether and how TREM2 shedding contributes to elevated TRLs is unknown. By complementary analyses of individuals with prediabetes and hepatic steatosis and preclinical models, we show that plasma sTREM2 levels correlate positively with plasma apolipoprotein C3 (APOC3), an apolipoprotein that slows TRL catabolism and predicts CVD risk. Individuals with prediabetes and hepatic steatosis had higher plasma concentrations of APOC3-rich TRLs 35 to 60 nm in diameter than healthy controls. Mouse models of prediabetes with hepatic steatosis revealed that the increased plasma concentrations of sTREM2, APOC3, and TRLs were due to activation of macrophage ADAM17, a TREM2 sheddase. Preserving macrophage full-length TREM2 protected against the elevated plasma APOC3, sTREM2, dyslipidemia, and atherosclerosis, whereas TREM2 deficiency increased APOC3, TRLs, and atherosclerosis. Mechanistically, full-length TREM2 mediates macrophage TRL uptake, preventing excessive hepatic APOC3-rich TRL release and atherosclerosis. Our findings identify macrophage TREM2 shedding as an upstream contributor to the elevated TRLs in hepatic steatosis, providing a mechanistic link between hepatic steatosis and CVD risk in prediabetes.

## Abstract 2

New findings reveal how fatty liver may disrupt immune cells that clear harmful blood fats, linking fatty liver to heart disease risk and potential treatments

## Graphical abstract

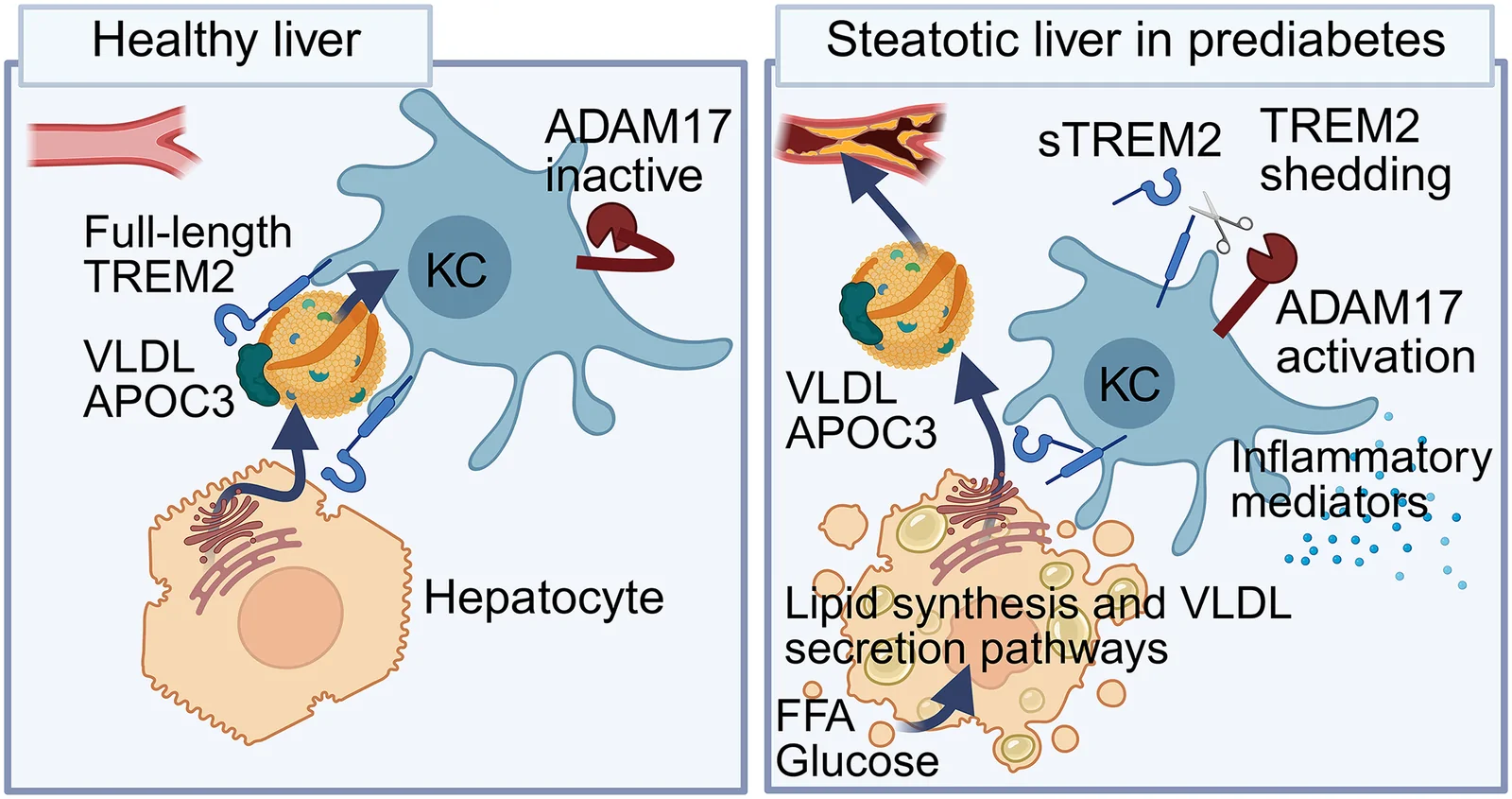

## Introduction

Metabolic dysfunction–associated steatotic liver disease (MASLD) is rapidly increasing and now has a global prevalence of over 30% by some estimates (1). This increase in MASLD is driven in part by the increasing prevalence of prediabetes and its progression to type 2 diabetes. MASLD is a risk factor for dyslipidemia characterized by elevated plasma triglycerides (TGs) and atherosclerotic cardiovascular disease (CVD) through mechanisms that are yet to be fully understood (2, 3). Plasma TGs are carried primarily by TG-rich lipoproteins (TRLs), which include VLDL secreted by the liver, chylomicrons secreted by the intestine, and their partly lipolyzed remnant lipoproteins (4). In addition to lipids, each TRL particle carries one molecule of the structural apolipoprotein B (APOB).

The recent studies showing that increased plasma levels of the cleaved extracellular domain of TREM2 (sTREM2; soluble triggering receptor expressed on myeloid cells 2) are associated with hepatic steatosis and steatohepatitis (5–7), symptomatic carotid stenosis, and atherosclerosis progression (8, 9) provide a potential clue to the mechanism behind the links among MASLD, prediabetes, and CVD risk. Full-length TREM2 is expressed by tissue macrophages (10) and activated hepatic stellate cells, but not by hepatocytes (11). TREM2 acts as a lipid sensor; its extracellular domain binds phospholipids and lipoproteins, which facilitates uptake of lipoproteins and extracellular lipid droplets, as well as efferocytosis of dead and dying cells (9, 12, 13). The extracellular domain of TREM2 is shed from the cell surface primarily by activation of the sheddase ADAM17 (a disintegrin and metalloprotease 17), generating sTREM2 (14).

TREM2 expression is increased in lipid-loaded macrophages (9, 15), and loss of TREM2 affects hepatic steatosis and atherosclerosis, both states characterized by the presence of lipid-loaded TREM2^+^ macrophages. Although the role of TREM2 in hepatic steatosis and atherosclerosis may depend on disease state, there is increasing evidence that macrophage expression of full-length (non-shed) TREM2 has beneficial effects due to efferocytosis and lipid sequestration both in the liver and in atherosclerosis.

For example, in one study, deletion of TREM2 in myeloid cells resulted in increased hepatic inflammation and fibrosis (13), with similar results obtained in whole-body TREM2-deficient mice (10), suggesting that myeloid cells are the main contributors to TREM2’s effects in the liver. In another study, TREM2 deficiency caused increased hepatic TG and cholesterol content and increased plasma lipid levels (16). In atherosclerosis models, myeloid cell– or hematopoietic cell–targeted TREM2 deficiency causes reduced lipid uptake and increased necrosis, attributed in part to reduced efferocytosis and increased cell death, which, depending on the model, can reduce the overall size of the lesion (17) while increasing necrotic cores (9).

Detrimental effects of TREM2 shedding are further supported by human studies showing that increased plasma levels of sTREM2 are associated with atherosclerosis progression (8, 9), increased mortality in individuals with preexisting coronary artery disease (18), and with CVD events in a cohort with preexisting ischemic stroke (19).

Apolipoprotein C3 (APOC3) is a small apolipoprotein that is associated with increased CVD risk, especially in individuals with diabetes and insulin resistance (20, 21). APOC3 controls clearance of TRLs by inhibiting the action of lipoprotein lipase, which mediates TG lipolysis (22), and by interfering with clearance of TRLs via hepatic LDL family receptors (23). There are no known links between APOC3 and TREM2.

Our results provide evidence of a link between macrophage TREM2 shedding, APOC3 and TRL levels, and atherosclerosis progression, with likely implications for CVD risk in individuals living with prediabetes and hepatic steatosis.

## Results

### Plasma sTREM2 is positively associated with APOC3 and increased concentrations of large to midsized TRL particles in individuals with MASLD and prediabetes.

We first investigated whether there is a link between plasma sTREM2 and APOC3 in individuals with MASLD in 3 cohorts (Supplemental Table 1; supplemental material available online with this article; https://doi.org/10.1172/jci.insight.202675DS1). Although there were differences in several parameters between the cohorts, such as age and BMI, sTREM2 levels correlated positively with APOC3, with 28% of variation in APOC3 explained by sTREM2 in individuals with MASLD (Figure 1A). Moreover, both sTREM2 and APOC3 correlated positively with plasma TG levels (*P* = 1.45 × 10^–4^, *r*^2^ = 0.49 and *P* = 1.46 × 10^–3^, *r*^2^ = 0.36, respectively).

We next investigated the content of APOC3 in TRL and LDL in a cohort closely matched for age, sex, and BMI; one group with normal intrahepatic TG content and normal glucose tolerance, and one group with elevated intrahepatic TG content and prediabetes (Supplemental Table 1, cohort 1). Individuals with prediabetes and elevated intrahepatic TG content exhibited higher APOC3 levels associated primarily with TRLs (Figure 1, B–E). There were no differences between the groups in TRL or LDL APOE, an apolipoprotein that facilitates hepatic clearance of TRLs. Moreover, the relative TRL content of APOB48 was higher in the group with elevated intrahepatic TG levels and prediabetes than in the group with normal liver TG content, consistent with increased levels of chylomicron-derived (APOB48-containing) TRLs (Figure 1C, insert). Therefore, TRLs in individuals with elevated liver fat and prediabetes are likely a mixture of VLDL and chylomicron remnants.

Next, we measured concentrations of size-defined TRL and LDL subpopulations by calibrated ion mobility analysis. Individuals with elevated intrahepatic TGs and prediabetes exhibited higher total concentrations of TRL particles (TRL-P) than individuals free of these conditions, with an increase primarily in the large to midsized TRLs of 35–45 nm and 45–60 nm diameter (Figure 1, F and G). There were no differences in LDL particle concentrations or size distributions (Figure 1, F and H).

Together, these results show that individuals with increased intrahepatic TG content and prediabetes have higher plasma concentrations of TRL particles in the large to midsized range and higher concentrations of sTREM2-, APOC3-, and APOC3-enriched TRLs.

### Plasma sTREM2 and APOC3 are increased in a mouse model of prediabetes and hepatic steatosis.

To investigate the mechanisms and consequences of the link between sTREM2 and APOC3, we used an LDL receptor–deficient (*Ldlr*^–/–^) mouse model of diet-induced prediabetes and hepatic steatosis. In this mouse model, like in humans (Figure 1A), a positive correlation between plasma sTREM2 and APOC3 was evident (Figure 2A). Compared with littermate controls fed a standard laboratory diet (SLD, chow diet), mice fed a diabetogenic diet with added cholesterol (DDC) exhibited elevated plasma sTREM2, APOC3, TGs, cholesterol, and free fatty acids (Figure 2, B–F). The elevated plasma lipids were due in part to an increased number of APOB-containing lipoprotein particles, as evidenced by the elevated levels of APOB and the large increase primarily in TRL particles and TRL remnants in plasma samples separated by size-exclusion chromatography (Figure 2, G and H). Consistently, ion mobility analysis demonstrated significant increases in TRL particle concentrations, particularly in TRL subpopulations in the 28 nm and 35 nm range (Figure 2I). Note that TRL sizes were smaller in mice than in humans.

Consistent with the existence of prediabetes, mice fed DDC were glucose intolerant compared with SLD-fed controls (Figure 2J). The liver phenotype was consistent with MASLD, with increased plasma aspartate aminotransferase (AST) levels indicating liver injury and elevated hepatic TGs with a nonsignificant (*P* = 5.53 × 10^–2^) increase in liver cholesterol (Figure 2, K–M). Moreover, hepatocytes exhibited increased lipid loading, measured by flow cytometry as BODIPY-positive hepatocytes (Figure 2N), and approximately 8% of hepatocytes were positive for 7AAD, indicating hepatocyte death (Figure 2O).

Liver bulk RNA-seq revealed increased expression of macrophage markers, such as *Cd68*, *C1qb*, and *C1qc*, with reduced expression of genes involved in cholesterol biosynthesis, such as *Cyp51*, *Hmgcr*, and *Fdft1* (Supplemental Figure 1). Increased hepatic inflammation and reduced cholesterol biosynthesis, indicative of increased ER cholesterol (24), were supported by gene set enrichment analysis (GSEA).

Flow cytometry was used to identify Kupffer cells (CD45^+^F4/80^hi^CD11b^Int^ CLEC4F^+^ cells) and infiltrated hepatic myeloid cells (CD45^+^F4/80^lo^CD11b^hi^CLEC4F^–^). A majority of immunoreactive TREM2 was localized at the cell surface in Kupffer cells (Figure 2P). Cell-surface TREM2 levels were reduced in Kupffer cells from DDC-fed mice, which contributed to a nonsignificant decrease in total cellular levels of TREM2. These results support increased TREM2 shedding in DDC-fed mice, consistent with the elevated plasma sTREM2. Moreover, the increased shedding of TREM2 was evident in the Kupffer cells and not in infiltrated myeloid cells (Figure 2Q).

Together, this mouse model of prediabetes and hepatic steatosis shows a positive correlation of plasma sTREM2 with APOC3, like in humans, and that the sTREM2 is derived, at least in part, from activated Kupffer cells.

### Macrophage full-length TREM2 lowers plasma TRLs by reducing APOC3 and APOB secretion rates and increasing TRL lipid clearance rates.

Next, to investigate whether macrophage TREM2 is directly responsible for regulating APOC3 and APOB levels, we analyzed apolipoprotein levels and kinetics in 3 *Ldlr*^–/–^ BM chimeras fed DDC: (a) *Trem2*^–/–^ hematopoietic chimeras (TREM2^H–/–^ mice), (b) chimeras deficient in ADAM17 in myeloid cells (ADAM17^M–/–^ mice), and (c) BM WT chimeras. Because ADAM17 is the principal TREM2 sheddase (14), ADAM17^M–/–^ mice have higher levels of myeloid full-length (non-shed) TREM2. Accordingly, plasma sTREM2 levels were lower in ADAM17^M–/–^ mice and were much lower in TREM2^H–/–^ mice than in *Ldlr*^–/–^ mice receiving WT BM (Figure 3A). Although the relative numbers of Kupffer cells (as a percentage of total hepatic leukocytes) did not differ between the groups, cell-surface TREM2 levels were markedly higher in Kupffer cells from ADAM17^M–/–^ mice than in both WT and TREM2^H–/–^ mice (Figure 3, B and C). Consistent with the increased ADAM17 activation and shedding of TREM2 in WT mice fed DDC, Kupffer cell surface TREM2 did not differ significantly between WT and TREM2^H–/–^ mice, suggesting that TREM2 shedding is nearly maximal in Kupffer cells in DDC-fed WT mice.

Macrophage TREM2 deficiency caused elevated plasma TG and cholesterol levels, as compared with myeloid ADAM17-deficient mice in which Kupffer cells carried higher levels of full-length TREM2 (Figure 3, D and E). *Ldlr*^–/–^ WT hematopoietic chimeras had plasma lipid levels similar to those of hematopoietic TREM2-deficient mice, in agreement with the lower levels of full-length TREM2 due to increased shedding in those mice. Cholesterol lipoprotein profiles revealed that TREM2^H–/–^ chimeras had higher TRL cholesterol and remnant/LDL cholesterol than myeloid ADAM17 chimeras and modestly higher TRL cholesterol than WT chimeras (Figure 3F). Thus, loss of macrophage TREM2, as compared with the increased levels of surface TREM2 in the ADAM17^M–/–^ mice, increases TRL and remnant/LDL cholesterol levels.

Plasma APOC3 levels were higher in TREM2^H–/–^ mice than in ADAM17^M–/–^ mice (Figure 3G). A similar pattern was observed for plasma APOB100, showing a higher concentration of APOB100 lipoprotein particles (VLDL, VLDL remnants, and LDL) in TREM2^H–/–^ mice versus ADAM17^M–/–^ mice (Figure 3H). No significant differences were observed in total APOB levels (Figure 3I). The increased plasma APOC3 levels were not due to increased hepatic transcription because hepatic *Apoc3* mRNA levels were decreased in TREM2^H–/–^ mice compared with WT chimeras (–0.47 log_2_ fold-change [log_2_FC] TREM2^H–/–^ versus WT; adjusted *P* = 0.011) and slightly increased in the ADAM17^M–/–^ mice (0.29 log_2_FC ADAM17^M–/–^ versus WT; adjusted *P* = 0.049). Therefore, increased levels of macrophage full-length TREM2 suppresses plasma APOC3 and APOB100 lipoprotein particles through a posttranscriptional mechanism.

Hematopoietic WT chimeras and TREM2^H–/–^ mice showed hepatic lipid vacuoles and elevated plasma AST levels, while ADAM17^M–/–^ mice were protected (Figure 3, J and K). These results are consistent with the interpretation that preserving full-length TREM2 on Kupffer cells (and likely on other macrophage populations) in the presence of hepatic steatosis and prediabetes suppresses both hepatic injury and APOC3 and APOB100 lipoprotein concentrations.

To gain mechanistic insight, bulk RNA-seq was performed on the livers of these mice. RNA-seq confirmed that *Trem2* mRNA was the most significantly downregulated gene in the TREM2-deficient BM chimeras (–3.56 log_2_FC TREM2^H–/–^ versus WT; adjusted *P* = 5.47 × 10^–18^; see Supporting Data Values, tab Figure S2A). Comparisons of transcriptomic signatures in livers of TREM2^H–/–^ mice (lack of macrophage TREM2) with ADAM17^M–/–^ mice (increased levels of full-length non-shed TREM2) by GSEA and ingenuity pathway analysis (IPA, QIAGEN) revealed increased signatures of cholesterol biosynthesis, fatty acid/acyl-CoA metabolism, and COPII-mediated vesicle transport pathways involved in VLDL generation and secretion (Supplemental Figure 2, A–C). These results, together with other pathways significantly enriched in the TREM2^H–/–^ mice, such as fatty acyl-CoA biogenesis (adjusted *P* = 1.02 × 10^–3^, *z* score 1.67), glycerophospholipid biosynthesis (adjusted *P* = 1.29 × 10^–3^, *z* score 1.89), and triacylglycerol biosynthesis (adjusted *P* = 2.51 × 10^–3^, *z* score 2.45) suggest increased VLDL secretion in TREM2^H–/–^ mice. Notably, no significant transcriptomic signatures indicated increased apoptosis or reduced efferocytosis. These findings were consistent with the lack of differences in Kupffer cell efferocytosis, measured as CLEC4F/TUNEL double-positive cells in the liver of the different groups of mice (Supplemental Figure 3, A–C).

To confirm increased APOC3 and APOB secretion, we next performed APOC3 and APOB kinetics analyses by ^13^C_6_,^15^N_2_-lysine heavy isotope tracing. These studies revealed that loss of macrophage TREM2 significantly increased the APOC3 and APOB secretion rates, consistent with the transcriptomic signatures revealed by the RNA-seq analysis, without affecting APOC3 and APOB fractional clearance rates (Figure 3, L and M).

We reasoned that the reduced APOC3 in mice with increased macrophage surface TREM2 levels (caused by myeloid cell ADAM17 deficiency) would increase TRL lipid clearance, since APOC3 prevents hepatic TRL clearance (23). We therefore assessed TRL lipid clearance rates by injecting radiolabeled TRLs into WT and ADAM17^M–/–^ mice and following their clearance from plasma into tissues. These experiments revealed that higher levels of macrophage surface TREM2 resulted in a faster clearance rate of both TRL TGs and TRL cholesterol, with a significant increase in hepatic uptake (Supplemental Figure 4, A–D).

Together, these results demonstrate that macrophage full-length (non-shed) TREM2 causes lower plasma APOC3 and APOB100 levels due to slower APOC3 and APOB secretion rates, and that the lower plasma cholesterol and TGs in these mice are also explained by an increased hepatic uptake of TRL lipids.

### sTREM2 alone does not affect APOC3 and APOB100 levels.

We next increased sTREM2 levels by an adeno-associated virus (AAV) approach to investigate whether the lower plasma APOC3 and APOB100 levels in ADAM17^M–/–^ mice could be mediated by reduced levels of sTREM2, rather than by higher levels of full-length TREM2. Increased plasma levels of sTREM2 for 18 weeks did not alter plasma lipids, APOC3, APOB100, hepatic steatosis, AST levels, or atherosclerosis (Supplemental Figure 5, A–J), suggesting that increased levels of full-length macrophage TREM2, not sTREM2, regulate plasma APOC3 and APOB100.

Because TREM2 is also expressed in brain microglia, we tested whether DDC-fed ADAM17^M–/–^ mice showed signs of altered hypothalamic gene expression, and thus whether the lowered plasma APOC3 could be explained by central regulation. However, despite functional myeloid cell ADAM17 deficiency and reduced plasma APOC3 levels, there were no detected changes in the hypothalamic expression of genes associated with energy balance or inflammation (Supplemental Figure 6, A–D).

### Lack of macrophage TREM2 increases TG, APOC3, and APOB100 levels in conditioned media in macrophage-hepatocyte cocultures.

Because full-length TREM2 contributes to the ability of macrophages to take up lipids, we next investigated whether lack of macrophage cell surface TREM2 increases APOC3 and APOB100 levels derived from lipid-loaded hepatocytes, using coculture models of mouse primary macrophages and hepatocytes. Hepatocytes were freshly isolated from *Ldlr*^–/–^ mice, labeled with Hoechst 33342 (a live cell-permeable dye), and then cocultured with or without BMDMs from WT mice, ADAM17^M–/–^ mice, TREM2-deficient mice, or mice deficient in both TREM2 and ADAM17 (Figure 4A). Oleic acid was added to promote TG accumulation and mimic hepatic steatosis.

TREM2 cell-surface levels were higher in ADAM17-deficient macrophages than in WT macrophages and were predictably undetected in TREM2-deficient macrophages and macrophages deficient in both TREM2 and ADAM17 (Figure 4B). ADAM17-mediated shedding of cell-surface TREM2 was most marked in unpolarized BMDMs and in BMDMs activated by inflammatory stimuli but not in alternatively activated BMDMs (Supplemental Figure 7A), consistent with known activators of ADAM17 and the inflammatory environment in the liver of our mouse model. We found no evidence of shedding of MerTK, another ADAM17 substrate (25), under the conditions used in our study (Supplemental Figure 7, B and C).

In agreement with TREM2’s ability to mediate lipid uptake, ADAM17-deficient macrophages, which have higher levels of full-length TREM2 on their surface, were more lipid-loaded than WT macrophages, and TREM2-deficient macrophages were less lipid loaded (Figure 4C). Macrophages deficient in both TREM2 and ADAM17 were also less lipid-loaded than both WT and ADAM17-deficient macrophages, in agreement with the interpretation that ADAM17 mediates its effects on lipid uptake primarily by reduced TREM2 shedding.

Coculture of hepatocytes with either WT or ADAM17-deficient BMDMs suppressed the oleic acid–induced TG, APOC3, and APOB100 levels in conditioned media, whereas coculture with BMDMs lacking TREM2 or both TREM2 and ADAM17 did not result in suppression of oleic acid–induced TG, APOC3, or APOB100 (Figure 4, D–F). Total conditioned media APOB levels did not change significantly with oleic acid incubation (Supplemental Figure 7D), which was expected (26) because APOB100 is more sensitive to the lipid-loaded status of the hepatocyte than APOB48 (27). Note that mouse hepatocytes secrete both APOB100 and APOB48 lipoprotein particles.

To investigate whether these effects of macrophage TREM2 require close proximity between hepatocytes and macrophages, similar coculture experiments were performed in which the hepatocytes and macrophages were physically separated using a Transwell system. Neither hepatocyte lipid loading nor TG, APOC3, or APOB100 secretion were affected by macrophage TREM2 levels when the macrophages were physically separated from the hepatocytes (Supplemental Figure 7, E–H).

Although we did not observe evidence for efferocytosis being affected by TREM2 in vivo, the cell culture system provided an ideal model to quantify macrophage efferocytosis of Hoechst 33342–labeled hepatocytes under controlled conditions. We found that lipid-loaded hepatocytes were engulfed by WT BMDMs at higher levels than hepatocytes not exposed to oleic acid, measured by cells double-positive for the macrophage marker CD115^+^ and Hoechst 33342 as a percentage of total Hoechst 33342^+^ cells (Supplemental Figure 8A). Moreover, ADAM17-deficient macrophages were more effective than WT macrophages at efferocytosis, and macrophages deficient in TREM2 or deficient in both ADAM17 and TREM2 were less effective at efferocytosis than WT macrophages, demonstrating that part of ADAM17’s efferocytotic effect is mediated by TREM2 under these conditions. There was a small additional efferocytosis effect in the TREM2-ADAM17 double-deficient macrophages, which was expected because of other known ADAM17 substrates involved in efferocytosis, such as MerTK. However, we did not find evidence for reduced MerTK shedding in the absence of myeloid ADAM17 (Supplemental Figure 7, B and C).

To confirm the role of full-length TREM2 in suppressing APOC3 and APOB100 in vivo, we fed *Ldlr*^–/–^ mice transplanted with (a) BM from WT (*Lyz2^Cre/Cre^*) mice, (b) BM from ADAM17^M–/–^ mice, or (c) BM from mice deficient in both myeloid ADAM17 and TREM2 (ADAM17^M–/–^ Trem2^–/–^) DDC for 18 weeks. Plasma sTREM2 levels were highest in WT mice and lowest in the ADAM17^M–/–^ TREM2^H–/–^ mice, as expected (Figure 4G). The lower plasma APOC3 and APOB100 levels in ADAM17^M–/–^ mice were prevented by deleting TREM2, supporting the conclusion that TREM2 is the main ADAM17 substrate mediating its effects on APOC3 and APOB100 lipoproteins (Figure 4, H and I). A subsequent validation experiment using the 4 groups of *Ldlr*^–/–^ mice transplanted with (a) BM from WT (Lyz2^Cre/Cre^) mice, (b) BM from ADAM17^M–/–^ (Lyz2^Cre/Cre^) mice, (c) BM from TREM2^H–/–^ (Lyz2^Cre/Cre^) mice, or (d) BM from mice deficient in both myeloid ADAM17 and TREM2 (ADAM17^M–/–^ TREM2^H–/–^ Lyz2^Cre/Cre^) on DDC for 12 weeks confirmed these observations and showed no significant differences in plasma APOC3, APOB100, cholesterol, or TGs between WT, TREM2^H–/–^, and ADAM17^M–/–^ TREM2^H–/–^ mice, with lower levels in ADAM17^M–/–^ mice (Supplemental Table 2).

Together, these results strongly support the proposal that although TREM2 can mediate efferocytosis, the main in vivo effect of TREM2 on TRLs in our models is unlikely to be due to differences in efferocytosis. Instead, macrophage full-length TREM2 mediates a reduction in APOC3 and APOB100 secretion by uptake of TRLs.

### TREM2 is a macrophage TRL receptor.

To directly test whether TREM2 mediates TRL uptake into macrophages, we incubated BM-derived macrophages from WT, ADAM17-deficient, TREM2-deficient, and ADAM17-TREM2 double-deficient mice with VLDL labeled with 1,1′-dioctadecyl-3,3,3′,3′-tetramethylindocarbocyanine perchlorate (DiI). These experiments revealed that ADAM17-deficient macrophages with higher levels of membrane full-length TREM2 had an increased ability to take up DiI-labeled VLDL, measured as uptake of both the DiI and APOB, whereas macrophages deficient in TREM2 or deficient in both TREM2 and ADAM17 had a markedly reduced ability to do so (Figure 5, A–C). The uptake was time dependent (Figure 5, B and C) and temperature dependent (no DiI-VLDL uptake was observed at 4°C), showing that the process required internalization of the TRL particles.

To provide additional in vivo support for this mechanism, we used IHC to show higher PLIN2 (a marker of lipid droplets) colocalization with CLEC4F (the Kupffer cell marker) in ADAM17^M–/–^ mice than in mice deficient in both ADAM17 and TREM2, without changes in the total CLEC4F area between groups (Figure 5, D–F). Moreover, some of the APOC3 immunoreactivity was colocalized with Kupffer cells (Supplemental Figure 8B), further supporting uptake by Kupffer cells.

### Macrophage full-length TREM2 prevents atherosclerosis progression.

We next analyzed the effects of macrophage full-length TREM2 on atherosclerosis in *Ldlr*^–/–^ mice transplanted with BM from WT, TREM2-deficient, or ADAM17^M–/–^ mice. Consistent with the changes in plasma lipids, APOC3, and APOB100 in these mice (Figure 3), loss of hematopoietic TREM2 led to increased aortic sinus atherosclerosis and necrotic core expansion, while preservation of full-length TREM2 by myeloid ADAM17 deficiency reduced atherosclerosis and necrotic cores (Figure 6, A–D).

To verify that the effects of myeloid cell–targeted ADAM17 deficiency were indeed mediated by increased levels of cell-surface TREM2, we transplanted *Ldlr*^–/–^ mice with BM from WT mice, ADAM17^M–/–^ mice, or mice lacking both ADAM17 and TREM2 in myeloid cells. These experiments confirmed that the protective effects of myeloid ADAM17 on both lesion size and necrotic core area were prevented when these cells also lacked TREM2 (Figure 6, E–G). These results strongly suggest that full-length (non-shed) TREM2 protects against atherosclerosis and necrotic core expansion in this mouse model. Consistent with the lack of significant differences in efferocytosis in the liver, no differences in efferocytosis were detected in aortic sinus lesions between the groups (Supplemental Figure 8, C–E).

## Discussion

Our findings demonstrate the existence of a mechanistic link between macrophage TREM2 shedding and increased concentrations of plasma APOC3 and APOB100 lipoprotein particles (VLDL, VLDL remnants, and LDL) due to increased hepatic release rates under conditions of prediabetes and hepatic steatosis with inflammation, with likely implications for dyslipidemia and the increased CVD risk associated with these conditions. In a healthy state, lipid-associated macrophages, including Kupffer cells, express full-length (membrane-associated non-shed) TREM2, which enables them to clear hepatic APOB100 lipoproteins that have not yet escaped into the bloodstream. Under these healthy conditions, full-length TREM2 is preserved because ADAM17 is largely inactive (Figure 6H).

With worsening insulin resistance, insulin’s antilipolytic effect in adipocytes is impaired, causing elevated release of fatty acids and increased uptake of these fatty acids by the liver. At the same time, elevated glucose levels fuel hepatic de novo lipogenesis. As hepatic steatosis develops, immune cells infiltrate the liver, resulting in increased inflammatory signaling in hepatocytes, which causes increased VLDL release (28). ADAM17 is activated in response to the inflammatory environment in Kupffer cells and likely other macrophage populations, and the extracellular domain of TREM2 is cleaved, generating increased sTREM2 in plasma while simultaneously reducing macrophage full-length functional TREM2 and impairing the macrophages’ capacity to clear TRLs secreted by the steatotic hepatocytes (Figure 6H). As a result, and likely because some of the secreted APOB100 lipoproteins undergo reuptake into hepatocytes, the liver further increases lipid metabolic pathways and secretion of APOC3 and VLDL, which serves to increase TG output.

How does macrophage TREM2 regulate hepatocyte VLDL and APOC3 secretion? The coculture studies showed that close proximity between the 2 cell types is needed. This could indicate that TREM2 is more effective in mediating uptake of TRLs when the macrophage is in close proximity to the hepatocyte, perhaps positioning TREM2 for optimal lipid particle uptake. Another possibility is that some of the effects of full-length TREM2 on suppressing APOC3 and APOB100 secretion is due to efferocytosis of lipid-loaded hepatocytes. Although TREM2-mediated efferocytosis was observed in the cell culture system, there was no strong evidence for differences in efferocytosis in vivo, either in the transcriptomics data or when TUNEL-positive Kupffer cells in the liver were quantified. We therefore favor the interpretation that TREM2’s effects on APOB100 and APOC3 secretion are primarily due to macrophage TRL uptake prior to TRLs entering the circulation. Indeed, VLDL uptake was markedly suppressed in macrophages deficient in TREM2 and increased in ADAM17-deficient macrophages with higher levels of full-length membrane TREM2. Macrophages deficient in both ADAM17 and TREM2 had a strikingly low TRL uptake similar to that of TREM2-deficient macrophages, indicating that TREM2 is the main ADAM17 substrate mediating the effects on TRL uptake. Moreover, a similar pattern was evident when lipid droplets in Kupffer cells in vivo were assessed by PLIN2 immunostaining, and some of the APOC3 immunoreactivity was colocalized with Kupffer cells in the liver. These observations strongly support the proposal that macrophage TREM2 is a TRL receptor that undergoes shedding in prediabetic hepatic steatosis.

Our findings may have important implications for understanding CVD risk and treatment options for individuals living with prediabetes and hepatic steatosis. We found that in individuals with prediabetes and MASLD, plasma levels of APOC3 and sTREM2 show a positive correlation. Moreover, these individuals have increased levels of TRLs in the large to midsize range, with TRL enriched in APOC3. Increased VLDL secretion likely contributes to this phenotype, but TRL remnants derived from chylomicrons, as evidenced by their APOB48 cargo, are also elevated, perhaps because of competition between VLDL-derived remnants and chylomicron-derived remnants for hepatic receptor-mediated clearance. Overall, these findings suggest a potentially novel mechanistic link between MAFLD and CVD risk.

Elevated APOC3 serum levels predict incident CVD in individuals with diabetes and insulin resistance (20, 21). Our previous studies show that APOC3 silencing reduces atherosclerosis in mouse models of type 1 diabetes and type 2 diabetes (21, 29, 30). Our findings strongly indicate that full-length cell-surface macrophage TREM2 suppresses atherosclerosis as well as the extent of lesion necrosis in the model of prediabetes and hepatic steatosis. Although macrophage TREM2 may have local effects in the lesion, lower levels of APOC3 and APOB100 lipoprotein particles are likely to contribute to the atheroprotective effects of full-length TREM2 in this model and to explain why some studies have not observed a protective effect of TREM2 on lesion size (9, 17).

Our study also has some limitations. Although TREM2 levels were higher in Kupffer cells than in other liver myeloid cell populations, we cannot rule out that other myeloid cells, including in other tissues, contribute to the APOC3 and TRL phenotypes. Moreover, ADAM17 has a multitude of substrates. Our data showing that mice deficient in hematopoietic TREM2 had similar phenotypes as mice with both myeloid-targeted ADAM17 and hematopoietic TREM2 deficiency suggest that the main effect of ADAM17 on APOC3 and TRLs is mediated by TREM2 shedding, but it is possible that other substrates contribute to the effects. Moreover, sTREM2 was expressed in hepatocytes in our study to achieve long-term increases in plasma sTREM2 levels (from 78 to 109 ng/mL). This increase did not affect plasma lipids, APOC3, APOB100, or atherosclerosis. It is possible that higher levels of sTREM2 or sTREM2 expressed under other cell type–selective promoters would have caused significant effects given that higher concentrations (300 ng/mL) of sTREM2 have been found to increase inflammatory gene expression in endothelial cells (31).

Together, our study demonstrates the existence of a potentially novel mechanistic link between macrophage TREM2 shedding and increased secretion of APOC3 and APOB100 lipoprotein particles, with likely implications for dyslipidemia and CVD risk in the setting of prediabetes with hepatic steatosis.

## Methods

### Sex as a biological variable.

Studies involving humans included both men and women. For the mouse studies, only male mice were used because the prediabetes phenotype in the diet-induced model we used is weaker in female mice (32). However, the findings are expected to be relevant for both sexes (32).

### Human studies.

Plasma samples were collected from men and women with obesity who had normal liver fat (<5%) and elevated liver fat (>10%) or histologically diagnosed MASLD at Washington University in St. Louis, Missouri, and the Veterans Affairs hospital in Seattle, Washington, in accordance with approved protocols and data access policies. In cohort 1 (Supplemental Table 1), liver fat content was measured by using MRI-proton density fat fraction (3-T superconducting magnet, Siemens). In cohort 2 and cohort 3, MASLD was identified by histological assessment of liver biopsies by an experienced hepatopathologist.

Fasting plasma APOC3 and sTREM2 levels were measured by ELISAs (RayBiotech ELH-ApoC3 and ELH-Trem2, respectively) according to the manufacturer’s protocols.

### Mice.

*Ldlr*^–/–^ mice and *Trem2*^–/–^ mice were purchased from The Jackson Laboratory. Mice with myeloid cell–targeted deletion of ADAM17 were generated by crossing *Adam17^fl/fl^* mice to mice expressing Cre recombinase under the control of the Lyz2 promoter (Lyz2^Cre/Cre^), generating *Adam17^fl/fl^ Lyz2^Cre/Cre^* mice (ADAM17^M–/–^) and *Adam17^WT/WT^* *Lyz2^Cre/Cre^* controls (WT). *Trem2*^–/–^ Adam17^M–/–^ mice (*Trem2*^–/–^ *Adam17^fl/fl^ Lyz2^Cre/Cre^*) were generated by breeding. WT controls (*Trem2^WT/WT^* Lyz2^Cre/Cre^), ADAM17^M–/–^, *Trem2*^–/–^, and ADAM17^M–/–^ *Trem2*^–/–^ mice were generated as breeding littermates. Because Cre homozygosity was needed to effectively delete myeloid ADAM17, we were careful to use Lyz2^Cre/Cre^ mice as controls.

Hematopoietic chimeras were generated by repopulating 12-week-old lethally irradiated *Ldlr*^–/–^ recipients with 5 *×* 10^6^ BM cells injected retro-orbitally from the above donor mice. After engraftment for 7 weeks, the hematopoietic chimeras were fed a high-fat, high-sucrose diet with added cholesterol (DDC; 36% fat, 36.2% sucrose, 0.15% added cholesterol; Bio-Serv, F4997, Frenchtown) (32) for 12–22 weeks. Some mice were fed an SLD. As an alternative to BM transplantation into *Ldlr*^–/–^ mice, in some experiments (Supplemental Figure 4) mice were treated with a liver-targeted GalNAc-modified *Ldlr* antisense oligonucleotide (ASO) or a control GalNAc ASO injected i.p. once a week for the duration of the study at 5 mg/kg/week.

### Liver-targeted expression of sTREM2.

A liver-targeted AAV (AAV8 containing the liver-specific human thyroxine binding globulin [TBG] promoter) was generated to express the extracellular portion (exon 1–2 and part of exon 3) of TREM2 (sTREM2), as described (33). An empty TBG-AAV8 was used as a control. The TBG-AAV8s were injected via the retro-orbital sinus at a titer of 1 × 10^11^ viral particles in 100 μL PBS/mouse. Two weeks later, the mice were fed DDC for 18 weeks.

### TRL and LDL particle concentrations, TRL lipid clearance, and apolipoprotein kinetics.

Differential ion mobility analysis was used to determine LDL and TRL particle concentrations in plasma, as described (34). Briefly, LDL was isolated at a density of 1.08–1.019 g/mL, whereas TRLs were isolated at a density of less than 1.019 g/mL. LDL and TRL particle concentrations (LDL-P and TRL-P) were calibrated and validated using external well-characterized plasma samples.

Clearance of radiolabeled chylomicrons was measured in mice fasted for 4 hours before injection of radiolabeled chylomicron via the retro-orbital sinus, as described previously (35).

Mouse apolipoprotein fractional clearance and secretion rates were quantified by stable isotope labeling by ^13^C_6_,^15^N_2_-lysine (Lys8), as described previously (36). Lys8 enrichment curves are shown in Supplemental Figure 9. Peptides measured are shown in Supplemental Table 3.

### Analysis of mouse plasma by ELISA and size exclusion chromatography.

Plasma lipoprotein profiles were analyzed by fast protein liquid size exclusion chromatography (34). Cholesterol content in each fraction was measured by a colorimetric assay (StanBio). Plasma apolipoprotein and liver enzyme quantification was performed with ELISAs: AST (ab263882, Abcam), APOB100 (ab230932, Abcam), TREM2 (ELM-TREM2, RayBiotech), and MerTK (ab210572, Abcam) according to the manufacturers’ protocols. The mouse APOC3 ELISA was established in-house using a capture and detection antibody pair from Abcam (ab241863) and the biotin conjugate kit (ab201795) to biotinylate the detection antibody. A quantitative mouse total APOB ELISA was developed in-house using an APOB N-terminal–specific antibody 2G11 (37) (MABS2046, Sigma-Aldrich).

### Quantitative liquid chromatography–tandem mass spectrometry analysis by parallel reaction monitoring.

Plasma proteins and purified human APOB were denatured and digested as described (36). Peptide digests were analyzed on an EASY-nLC 1200 UHPLC system coupled to an Orbitrap Fusion Lumos Tribrid mass spectrometer (Thermo Fisher Scientific) (36). Data were analyzed in Skyline-Daily (v25.1.1.206) (38).

### Flow cytometry of primary hepatocytes and leukocytes.

Livers were digested with Liberase (05401127001, Sigma-Aldrich) perfusion and Percoll (17544502, Cytiva) purification (39), separating hepatocytes from non-parenchymal cells. Flow cytometry was performed on hepatocytes and non-parenchymal cells separately. Antibodies used for flow cytometry are shown in Supplemental Data 1. Fluorescence-minus-one controls (omitting one of the targeting antibodies at a time in the presence of all other antibodies) were used for gating strategies (Supplemental Figure 10).

### Liver histology and IHC.

Sections of FFPE mouse liver (10 µm) were stained with H&E for histology. Paraffin liver sections were deparaffinized and antigen retrieved by the citrate buffer and incubated with TUNEL reaction mixture (20 μL/section, in situ cell death detection kit TMR red, 12156792910, Roche Diagnostics) according to the manufacturer’s protocol. After washing, the sections were mounted with antifade mounting medium with DAPI (Vector Lab H-2000).

Goat anti-mCLEC4F antibody (4 μg/mL, AF2784, R&D Systems) or normal goat IgG (4 μg/mL, AB-108-C, R&D Systems) were used to stain Kupffer cells. Rabbit anti-ADRP/Perilipin2 antibody (1:300, 15294-1-AP, Proteintech), or Rabbit IgG (Proteintech, 15294-1), was used to stain lipid droplet particles. Anti-APOC3 rabbit serum (1:1,000, Ionis Pharmaceuticals) or normal rabbit serum were used to stain APOC3.

The secondary antibodies used were Alexa Fluor 647 donkey anti-goat IgG (1:500, A21447, Invitrogen), Alexa Fluor 555 goat anti-rabbit IgG (1:500, A21428, Invitrogen), or Alexa Fluor 555 goat anti-rabbit IgG. The sections were mounted with DAPI Fluoromount-G (0100-20, Southern Biotech), and signals were captured and quantified by Keyence BZ-X810 all-in-one fluorescence microscope.

Lipid vacuoles in mouse liver specimens were scored using ImageJ (NIH 1.54u8). H&E-stained slides (10×) were opened in ImageJ (NIH) and converted to 8 bits to set the threshold. Circular objects were defined between 0.85 and 1. After removing outliners according to a set parameter of 20/50, the remaining circles on the slide were converted into binary data for area counting. Slides were scored by 2 individuals blinded to the genotypes.

### Primary hepatocyte and Kupffer cell isolation and hepatocyte-macrophage coculture.

BMDMs were prepared as described (7). Primary hepatocytes were isolated from 6–8-week-old *Ldlr*^–/–^ mice as described above. Kupffer cells in the liver leukocyte fraction of the same mice were collected and further purified by gradient centrifugation and used in flow analysis with a panel of cell type–specific markers (Supplemental Data 1). To investigate TRL uptake, nonpolarized BMDMs differentiated in the presence of M-CSF for 6 days were incubated in RPMI medium without FBS overnight, and then treated with DiI-labeled human VLDL (Kalen Biomedical, 770130-9) at 50 μg/mL for 30 or 60 minutes at either 4°C or 37°C. Cells were fixed and APOB was detected using an APOB antibody (1:500, ab7616, Abcam, in 1 mg/mL BSA and 0.1% saponin) at 4°C overnight. Alexa Fluor 488 donkey anti-goat IgG secondary antibody (1:500, Invitrogen) was used to visualize the APOB signal. DiI, APOB, and nuclei (DAPI) signals were captured and quantified by Keyence BZ-X810 all-in-one fluorescence microscope. Representative images were captured by a Nikon A1R confocal microscope.

### Atherosclerosis analysis.

Aortic sinus atherosclerotic lesion areas and necrotic core areas were measured after Movat’s pentachrome staining at the indicated distances from the appearance of all 3 aortic valve leaflets, as described (40). Mac2-positive TUNEL-positive cells were analyzed by IHC, as described previously (41).

### Statistics.

Group sizes were determined by power calculations prior to the study. Littermate mice were randomized into groups based on similar body weights. All analyses were done in a masked fashion on coded samples. Statistical analyses were performed using GraphPad Prism 10 or R Studio or IPA (QIAGEN) for RNA-seq studies. We used 2-tailed unpaired Student’s *t* tests to compare differences between 2 groups when normal data distribution, assessed by D’Agostino-Pearson normality tests, was confirmed. To compare 3 or more groups, we used 1-way or 2-way ANOVA followed by post hoc tests. Statistical outliers were identified by the ROUT (Q = 0.1%) method. Data not normally distributed were analyzed by Kruskal-Wallis tests followed by Dunn’s multiple-comparison tests (multiple groups) or Mann-Whitney tests (2 groups). An adjusted *P* value of less than 0.05 was considered statistically significant. See Supporting Data Values.

### Study approval.

All mouse experiments were performed in accordance with an approved University of Washington IACUC protocol (no. 3154-01). In adherence to the Declaration of Helsinki, all participants provided written informed consent before participating in the study, which was approved by the IRBs of Washington University School of Medicine in St. Louis, Missouri, USA (no. 201111078 and 201512086) and the Veterans Affairs Puget Sound Health Care System, Seattle, Washington, USA (no. 01008). All samples were deidentified and analyzed in a blinded manner.

### Data availability.

Further details are provided in Supplemental Data 1 and Supporting Data Values. The liver bulk RNA-seq data have been deposited in NCBI’s Gene Expression Omnibus (GEO GSE343509 and GSE343510).

## Author contributions

JT designed and performed a majority of the experiments. KEB designed and directed the study. JEK, LJDH, and GNI provided additional direction. NOD, SK, and GIS provided plasma samples from study participants with MASLD and controls. JT and KEB wrote the manuscript. BS, ARK, HZ, JC, FK, MSA, JMF, JL, AT, MDD, CCH, TV, and BSJD performed experiments and analyzed data. AEM provided advice and reagents. All authors reviewed the manuscript and provided final approval for submission.

## Conflict of interest

AEM is employed by Ionis Pharmaceuticals and provided antisense oligonucleotides for the study. KEB serves on the Scientific Advisory Board of Esperion Therapeutics.

## Funding support

This work is the result of NIH funding, in whole or in part, and is subject to the NIH Public Access Policy. Through acceptance of this federal funding, the NIH has been given a right to make the work publicly available in PubMed Central.

- NIH grants R35HL150754, R35HL184266, and P01HL151328 (to KEB); R01DK135756 (to LJDH); R01DK121756 (to JEK); P30DK056341 (to Washington University Nutrition Obesity Research Center); and K01HL153205 and R21DK127296 (to MDD).
- Diabetes Research Center at University of Washington (P30DK017047).

## Supplementary Material

Supplemental data

Supplemental data 2

Supporting data values

## Figures and Tables

**Figure 1:**
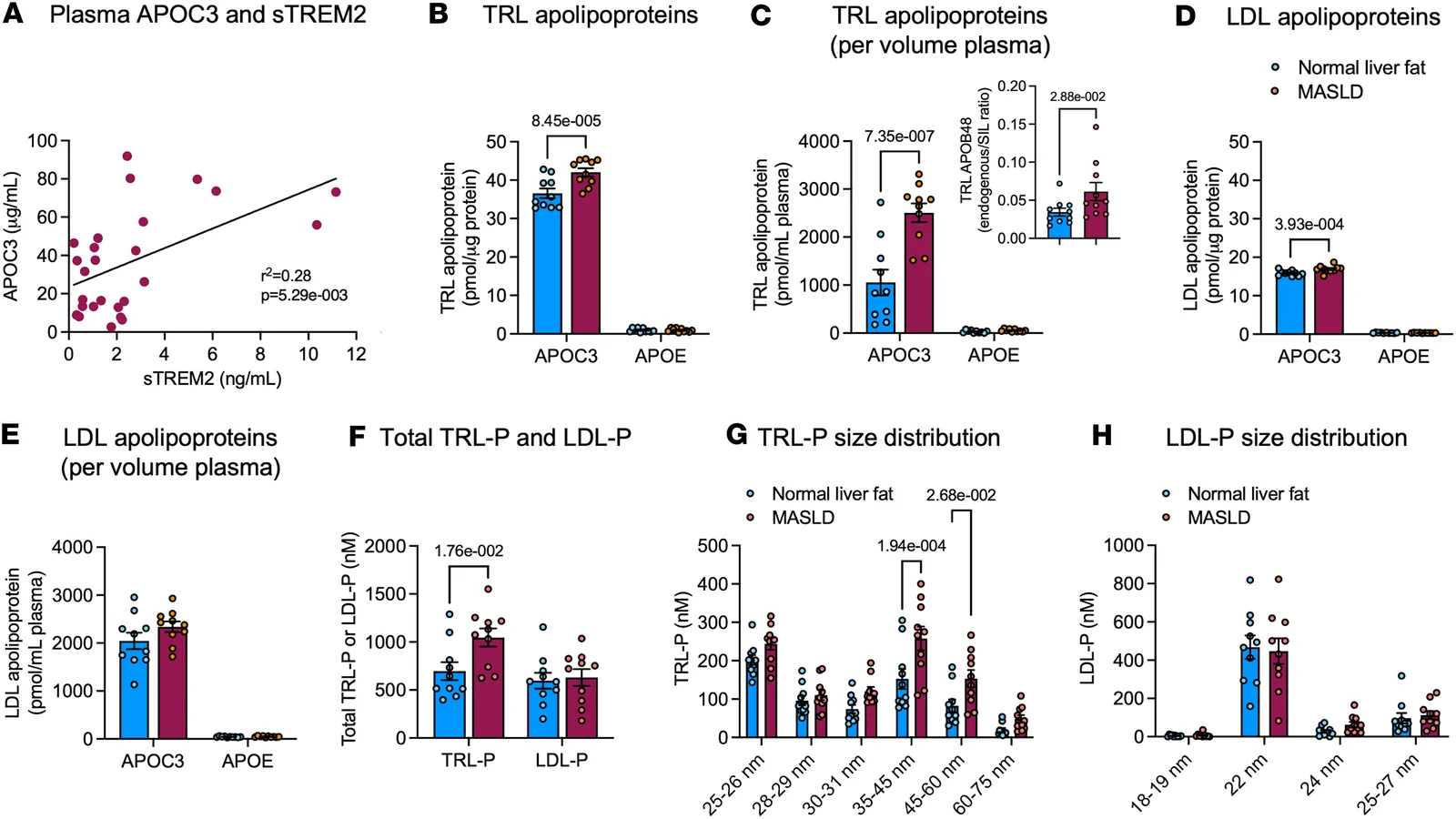
In individuals with MASLD and prediabetes, plasma sTREM2 is associated with plasma APOC3 and concentrations of APOC3-enriched TRL particles are increased. (**A**) Plasma samples from individuals with MASLD were analyzed for APOC3 and sTREM2 levels by ELISAs (cohorts 1–3; Supplemental Table 1). (**B** and **C**) Apolipoproteins in isolated TRLs (density < 1.019 g/mL) from individuals with normal liver fat (*n* = 10) versus individuals (*n* = 10) with glucose intolerance and high liver fat (MASLD) were analyzed by targeted mass spectrometry (cohort 1; Supplemental Table 1). Relative levels of APOB48 are shown as an insert in **C**. (**D** and **E**) Apolipoproteins in isolated LDL (density 1.08–1.019 g/mL) from individuals with normal liver fat (*n* = 10) versus individuals (*n* = 10) with glucose intolerance and high liver fat were analyzed by targeted mass spectrometry. (**F**) Total concentrations of TRL and LDL particles in the isolated density fractions were determined by calibrated ion mobility analysis. (**G**) TRL subpopulation particle size and concentrations (TRL-P) were measured by calibrated ion mobility analysis. (**H**) LDL subpopulation particle size and concentrations (LDL-P) were measured by calibrated ion mobility analysis. The 18–19 nm LDL population corresponds to small dense LDL. Mean ± SEM (**B**–**H**). Statistical analyses: Pearson’s correlation (**A**), 2-way ANOVA with Šídák’s multiple-comparison test (**B**–**H**). See Supporting Data Values for group sizes and statistics.

**Figure 2:**
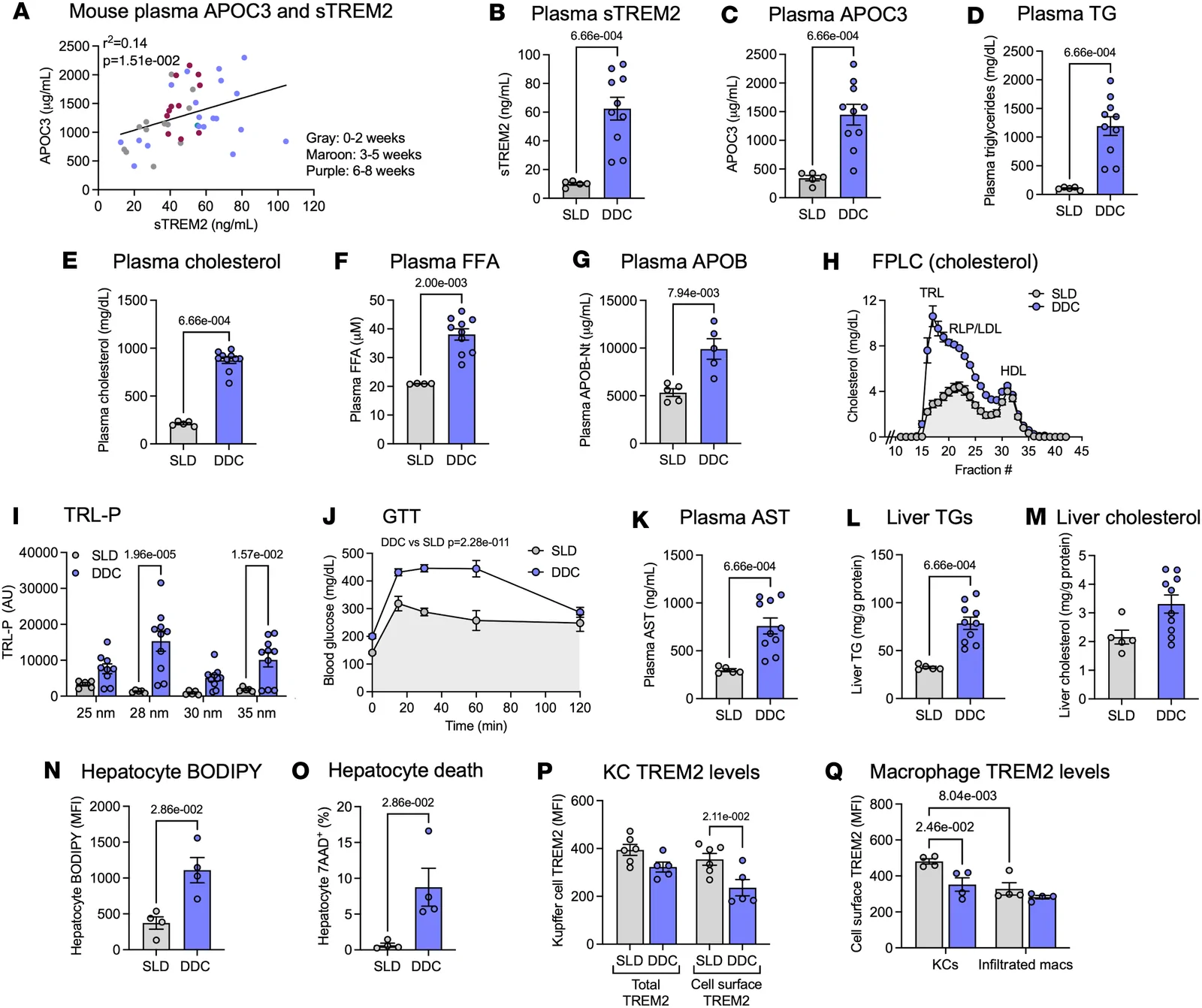
Plasma sTREM2 and APOC3 are increased in a mouse model of prediabetes and hepatic steatosis. (**A**) Plasma APOC3 and sTREM2 were measured by ELISAs in samples collected from *Ldlr*^–/–^ mice fed a diabetogenic diet (DDC) for 0–2 weeks (*n* = 16), 3–5 weeks (*n* = 15), and 6–8 weeks (*n* = 10). (**B**–**G**) Plasma sTREM2, APOC3, triglycerides, cholesterol, free fatty acids (FFAs), and total APOB (measured by an antibody that binds to the N-terminus; APOB-Nt) levels in *Ldlr*^–/–^ mice fed an SLD or DDC for 22 weeks. (**H**) Cholesterol lipoprotein profile at the 22-week time point. RLP, remnant lipoprotein particle. (**I**) TRLs were isolated from mouse plasma (d < 0.019 g/mL), and TRL subpopulation particle concentrations (TRL-P) and sizes were measured by ion mobility analysis. (**J**) Glucose tolerance tests at 20 weeks. (**K**) Plasma AST at 22 weeks. (**L**) Liver triglycerides. (**M**) Liver cholesterol. (**N** and **O**) Hepatocytes were isolated and analyzed for lipid loading (BODIPY) and viability (7AAD) by flow cytometry. (**P** and **Q**) Total TREM2 and cell surface TREM2 were measured by flow cytometry in permeabilized and non-permeabilized Kupffer cells (KCs), respectively, and infiltrated macrophages in the liver. Mean ± SEM (**B**–**Q**). Statistical analyses: Pearson’s correlation (**A**), Mann-Whitney (**B**–**G** and **K**–**O**), 2-way ANOVA with Šídák’s multiple-comparison test (**I** and **J**), 2-way ANOVA with Tukey’s multiple-comparison test (**P** and **Q**). See Supporting Data Values for group sizes and statistics.

**Figure 3:**
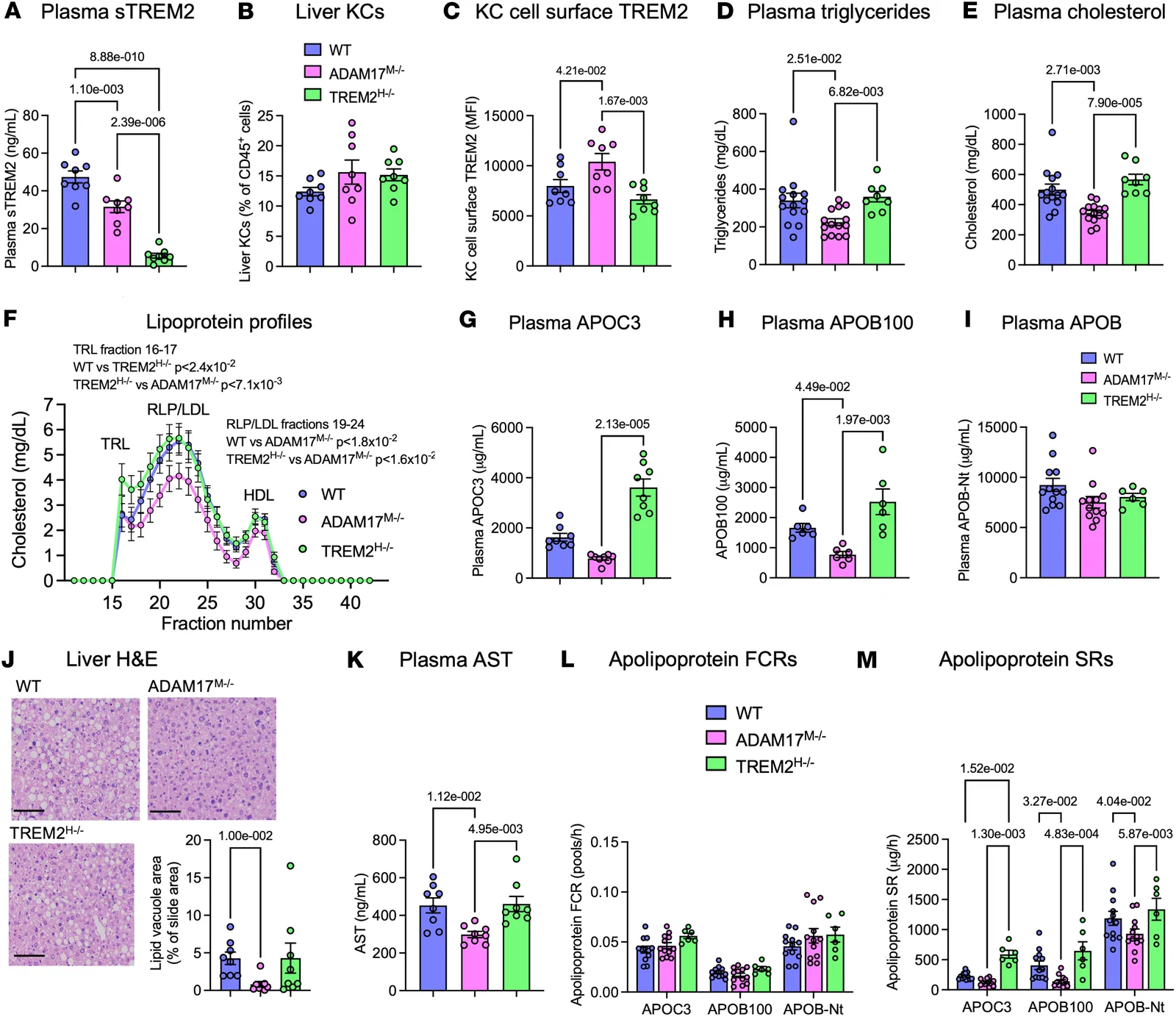
Macrophage full-length TREM2 lowers plasma TRLs by reducing APOC3 and APOB secretion rates. *Ldlr*^–/–^ mice were transplanted with BM from WT mice, myeloid *Adam17*^–/–^ mice, or *Trem2*^–/–^ mice. After a 7-week recovery period, the mice were fed DDC for 18 weeks. (**A**) Plasma sTREM2 levels were measured by ELISA at 18 weeks of DDC. (**B**) Liver Kupffer cells (KCs) were quantified as CLEC4F^+^ CD11b^+^ F4/80^hi^ cells as a percentage of total liver CD45^+^ cells by flow cytometry. (**C**) Cell-surface TREM2 levels (MFI) in the hepatic KC population. (**D** and **E**) Plasma triglyceride and cholesterol levels. (**F**) Cholesterol lipoprotein profiles. RLP, remnant lipoprotein particle. (**G**) Plasma APOC3 levels. (**H** and **I**) Plasma APOB100 and total APOB levels. (**J**) H&E-stained liver sections and quantification of lipid vacuolar area as a percentage of total analyzed slide area (1.92 mm^2^). Scale bar: 100 μm. (**K**) Plasma AST levels. (**L** and **M**) Apolipoprotein kinetics measured by targeted mass spectrometry after a bolus injection of Lys8. (**L**) Fractional clearance rates (FCRs). (**M**) Secretion rates (SRs). Statistical analyses: 1-way ANOVA with Tukey’s multiple-comparison test (**A**–**C**), Kruskal-Wallis with Dunn’s multiple-comparison test (**D**, **E**, and **G**–**K**), 2-way ANOVA with Tukey’s multiple-comparison test (**F**, **L**, and **M**). See Supporting Data Values for group sizes and statistics.

**Figure 4:**
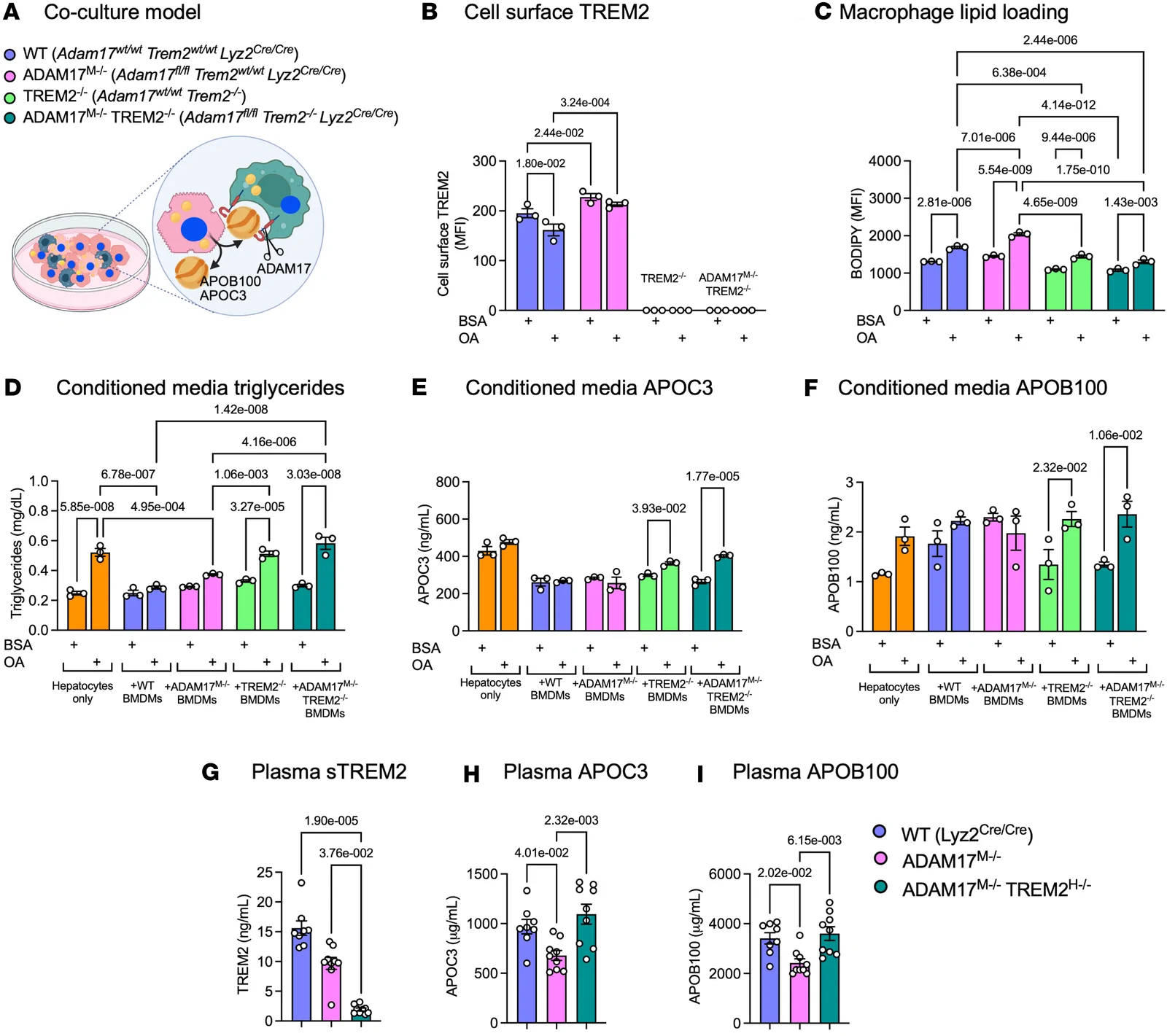
Lack of macrophage TREM2 interaction with hepatocytes drives triglyceride, APOC3, and APOB100 secretion from steatotic hepatocytes. (**A**) Coculture model. Blue nuclei indicate Hoechst 33342–labeled hepatocytes. (**B**–**F**) Hepatocytes were isolated from *Ldlr*^–/–^ mice labeled with Hoechst 33342 and immediately cocultured for 20 hours with and without BMDMs from WT mice, myeloid-targeted ADAM17^M–/–^ mice, *Trem2*^–/–^ mice, and mice deficient in both TREM2 and myeloid ADAM17 in the presence of oleic acid (OA) bound to BSA or with BSA alone. Cell-surface TREM2 (**B**) and lipid loading (BODIPY) of macrophages (**C**) were analyzed by flow cytometry. TGs, APOC3, and APOB100 in the conditioned media were measured by colorimetric assays and ELISAs (**D**–**F**). (**G**–**I**) *Ldlr*^–/–^ mice were transplanted with BM from WT mice, ADAM17^M–/–^ mice, and ADAM17^M–/–^ *Trem2*^–/–^ mice. After 7 weeks of recovery, the mice were fed DDC for 18 weeks, and plasma sTREM2, APOC3, and APOB100 were measured. Statistical analyses: 2-way ANOVA with Tukey’s multiple-comparison test (**B**–**D**), 2-way ANOVA with Šídák’s multiple-comparison test (**E** and **F**), Kruskal-Wallis with Dunn’s multiple-comparison test (**G** and **I**), 1-way ANOVA with Tukey’s multiple-comparison test (**H**). See Supporting Data Values for group sizes and statistics.

**Figure 5:**
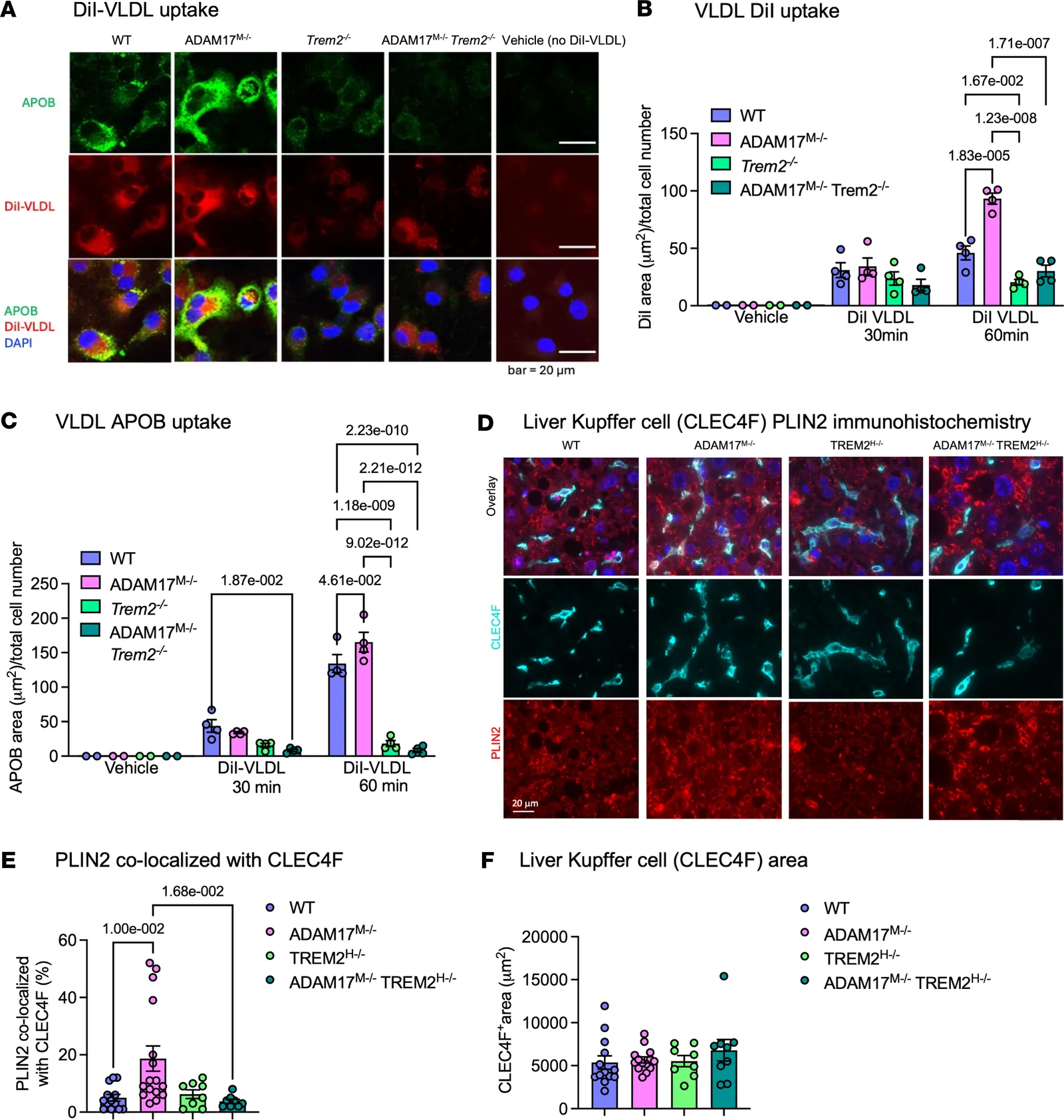
Macrophage TREM2 mediates TRL uptake. (**A**) BMDMs from the indicated genotypes were incubated in the presence of DiI-VLDL for 30 or 60 minutes or vehicle for 30 minutes. The cells were them immunostained with a goat anti-APOB antibody (Ab7616, 1:500 dilution) followed by a donkey anti-goat Alexa Fluor 488 antibody (green). Cell nuclei were stained with DAPI (blue) and DiI was visualized as red stain. Scale bar: 20 μm. (**B**) Quantification of DiI uptake/total cell number (nuclei). (**C**) Quantification of APOB uptake/total cell number (nuclei). Data are shown from 1 representative experiment repeated 3 times in independent cell isolates with similar results. (**D**) Liver sections from the 4 groups stained with antibodies against CLEC4F (Kupffer cell marker) and PLIN2 (lipid droplet marker). Scale bar: 20 μm. (**E**) Quantitation of PLIN2 colocalized with CLEC4F. (**F**) Quantification of CLEC4F area/45,000 μm^2^. Statistical analyses: 2-way ANOVA with Tukey’s multiple-comparison test (**B** and **C**), 1-way ANOVA with Tukey’s multiple-comparison test (**E**), Kruskal-Wallis with Dunn’s multiple-comparison test (**F**). See Supporting Data Values for group sizes and statistics.

**Figure 6:**
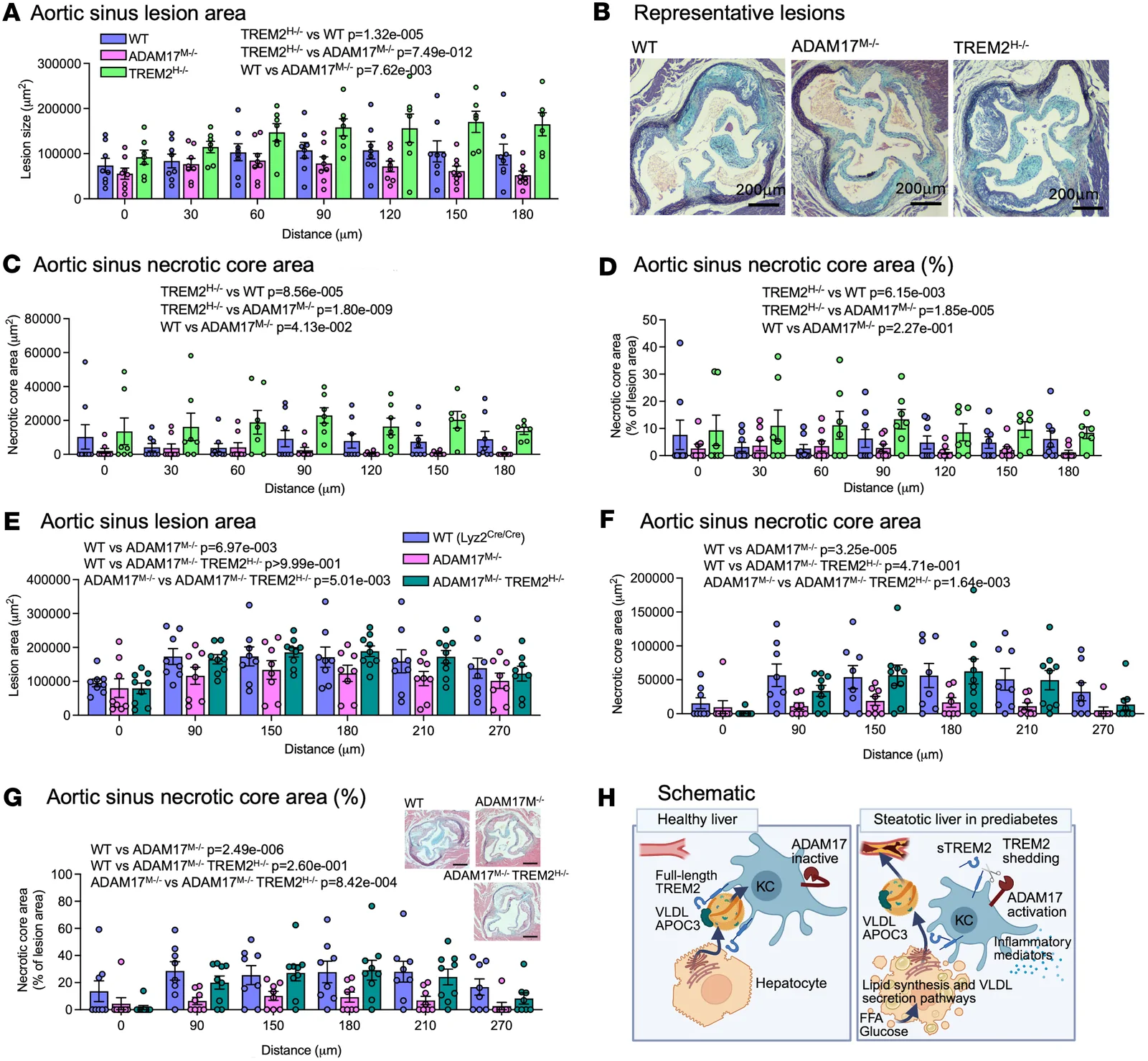
Full-length macrophage TREM2 protects against atherosclerosis. (**A**–**D**) *Ldlr*^–/–^ mice were transplanted with BM from WT mice, *Trem2*^–/–^ mice, or myeloid *Adam17*^–/–^ mice. After BM repopulation, the mice were fed DDC for 18 weeks. (**A**) Aortic sinus lesion area over distance from the appearance of all 3 leaflets. (**B**) Representative photos. Scale bar: 200 μm. (**C**) Lesion necrotic core area. (**D**) Lesion necrotic core area as a percentage of lesion area. (**E**–**G**) *Ldlr*^–/–^ mice were transplanted with BM from WT Lyz2^Cre/Cre^ mice, myeloid *Adam17*^–/–^ mice, or mice deficient in myeloid ADAM17 and TREM2. (**E**) Aortic sinus lesion area over distance from the appearance of all 3 leaflets. (**F**) Lesion necrotic core area. (**G**) Lesion necrotic core area as a percentage of lesion area and representative lesions. Scale bar: 200 μm. (**H**) Schematic showing full-length Kupffer cell (KC) TREM2 taking up excess lipid particles, such as APOC3-containing VLDL secreted from hepatocytes, in a healthy liver when ADAM17 is inactive (left), and TREM2 shedding due to inflammation and ADAM17 activation in a steatotic liver in prediabetes, resulting in reduced TREM2-mediated KC uptake of TRLs; an increase in cholesterol, TG, and phospholipid synthesis pathways and VLDL secretion pathways in hepatocytes; and an increase in hepatic VLDL-APOC3 secretion and atherosclerosis (right). Created using BioRender.com. Mean ± SEM. Statistical analyses: 2-way ANOVA with Tukey’s multiple-comparison test (**A** and **C**–**G**). See Supporting Data Values for group sizes and statistics.

## Version 1 08/27/2026

In-Press Preview

## Version 2 10/08/2026

Electronic publication
