# Supplementary material for: Macrophage TREM2 is a triglyceride-rich lipoprotein receptor that undergoes shedding in prediabetic hepatic steatosis
Source: JCI Insight. 2026 Aug 27;11(19):e202675. doi: 10.1172/jci.insight.202675 (PMC13726429; doi:10.1172/jci.insight.202675)
Supplement: Supplementary file 1 [file jciinsight-11-202675-s119.pdf]

Supplemental Materials

Macrophage TREM2 is a triglyceride-rich lipoprotein receptor that undergoes shedding in prediabetic hepatic steatosis

Jingjing Tang, Jenny E. Kanter, Baohai Shao, Masami Shimizu-Albergine, Farah Kramer, Ahreum Khang, Jason Luo, Huaqing Zheng, Alan Tran, Jocelyn Cervantes, Jeremy M. Frey, Mauricio D. Dorfman, Cheng-Chieh Hsu, Laura J. den Hartigh, Tomas Vaisar, Brandon S.J. Davies, Adam E. Mullick, George N. Ioannou, Gordon I. Smith, Samuel Klein, Nicholas O. Davidson, Karin E. Bornfeldt

Detailed Methods
Supplemental Table 1
Supplemental Table 2
Supplemental Table 3
Supplemental Figure 1
Supplemental Figure 2
Supplemental Figure 3
Supplemental Figure 4
Supplemental Figure 5
Supplemental Figure 6
Supplemental Figure 7
Supplemental Figure 8
Supplemental Figure 9
Supplemental Figure 10

Detailed Methods

Mice. All mice were on the C57BL/6J background and were littermates within each experiment. Mice were housed in an SPF facility with 12 h light-dark cycle, with food and water ad libitum.

Analyses of plasma lipids and TRL lipid clearance. Blood glucose, plasma triglycerides, and cholesterol were measured throughout each of the studies. Blood glucose was measured from the saphenous vein blood by stick tests (OneTouch Ultra). Plasma cholesterol and triglyceride levels were determined by Cholesterol Liquicolor and Triglyceride Liquicolor, respectively (Stanbio, purchased via Fisher Scientific). Plasma free fatty acids were assayed by a colorimetric kit (Abcam, #ab282927).

Clearance of radiolabeled chylomicrons was measured in mice fasted for 4 h before injection of radiolabeled chylomicron via the retro-orbital sinus, as described previously (reference 35 – main manuscript). The radiolabeled chylomicrons were generated from fasted *Gpihbp1*$^{-/-}$ mice orally gavaged with olive oil mixed with [9,10-^{3}H(N)]-Triolein (Perkin Elmer, NET431001MC) and [4-^{14}C]-Cholesterol (Perkin Elmer, NEC018050UC). The radio-labeled chylomicrons were resuspended in sterile PBS for injection. After chylomicron injection into experimental mice, blood was collected at 1, 5,

15, and 30 minutes, and radioactivity was measured by scintillation counter. Different organs were collected at the end point for analysis of radioactivity.

Apolipoprotein kinetics studies. Mouse apolipoprotein fractional clearance and secretion rates were quantified by stable isotope labeling by $^{13}C_6$, $^{15}N_2$-Lysine (Lys8), as described previously (reference 36 – main manuscript). In short, Lys8 was injected retro-orbitally at 500 μmol/kg body weight, and blood samples (15 μL) were collected at 2, 4, 6, 8, 10, 20, 24, 28, 32 h after injection. Targeted liquid chromatography tandem mass spectrometry was used to detect Lys8 enrichment in APOC3 and APOB peptides (**Supplemental Table 1**). The fractional clearance rate (FCR) for APOB (peptides common to APOB100 and APOB48), APOB100 (peptides specific to APOB100), and APOC3 was calculated as the monoexponential decay rate of the tracer enrichment, and secretion rates (SRs) were determined as the product of the plasma concentration of each apolipoprotein measured by ELISA and the respective average FCR for all measured peptides in that apolipoprotein and adjusted for blood volume estimated by body weights. Time points that fell before the peak H:L value were eliminated (i.e., the protein turned over too slowly for the sampling schedule such that early time points had not achieved peak enrichment), and late time points that did not fall on the log-linear portion of the curve were not included in the monoexponential slope analysis. When the linear regression resulted in an r^2 <0.86, data points that caused the regression line r^2 to be <0.86 were sequentially eliminated (see Supporting Data Values).

Glucose tolerance tests. Mice were fasted for 4-6 hours before injecting 0.75-1.0 mg/g body weight of glucose intraperitoneally. Blood glucose was monitored at 0, 15, 30, 60 and 120 minutes after glucose administration.

Quantification of total mouse APOB. Because no quantitative ELISA for mouse total APOB was available, we developed a standard for the APOB ELISA by a targeted mass spectrometry approach. Plasma samples were coated onto 96-well high-binding plates overnight (4°C), then biotinylated 2G11 (an APOB N-terminal-specific antibody) was added to the wells after blocking with PBS/1% BSA/10% FBS. A pooled mouse plasma sample (a mixture of SLD and DDC-fed samples) with APOB determined by mass spectrometry was used as standard.

Absolute concentrations of total APOB (APOB-Nt) and APOB100 in the pool of mouse plasma were determined by two complementary strategies: (1) a peptide-based calibration using custom-made synthetic Lys8-labeled mouse APOB peptides (Biosynth International., Inc., Gardner, MA) and (2) protein-based calibration using full-length human APOB purified from human plasma (Innovative Research, purity: > 98% by SDS-PAGE; SKU:IHUAPOB100UG, 1 mg/mL). Surrogate Lys8-peptides for total mouse APOB were GFEPTLEALFGK and INIDIPLPLGGK; surrogate Lys8-peptides for mouse APOB100 were FFLPDFK and SLPVGNTVFDLNK. This approach was possible because human and mouse APOB share extensive sequence homology, including multiple conserved tryptic peptides that are detectable by mass spectrometry. Thus, the use of human APOB provides a suitable standard for peptide identification and quantification.

For the peptide standard approach, the surrogate synthetic peptides for total APOB and APOB100 (0.5–16 nM) were spiked into mouse plasma before digestion, the peak areas of light (endogenous) and heavy (Lys8-labeled) peptides were measured and the heavy to light ratios were calculated. The calibration curves were generated by plotting [H:L ratio] versus [nM]. Calibration curves (0.5–16 nM) showed excellent linearity. For Lys8-GFEPTLEALFGK, the curve yielded r^2=0.9990, slope=1.1447, intercept=0.0185; INIDIPLPLGGK, r^2=0.9963, slope=0.7856, intercept= –0.0045; for Lys8-FFLPDFK, r^2=0.9981, slope=1.9575, intercept= −0.0407; and for Lys8-SLPVGNTVFDLNK, r^2=0.9998, slope=1.0682, intercept=0.0105. Results were reproducible in duplicate assays (maximum CV% < 7.2%). Calculated endogenous concentrations at H:L=1 were: GFEPTLEALFGK 0.8574 pmol/150 µL and INIDIPLPLGGK 1.2785 pmol/150 µL for total APOB; and FFLPDFK 0.5317 pmol/150 µL and SLPVGNTVFDLNK 0.9263 pmol/150 µL for APOB100. Importantly, GFEPTLEALFGK is a shared peptide between human and mouse APOB and the value from Lys8-lebeled peptide was in closest agreement with the protein standard approach (standard-addition using purified full-length human APOB; 0.6657 pmol/150 µL), whereas INIDIPLPLGGK gave a systematically higher value. We therefore selected Lys8-GFEPTLEALFGK as the reporting peptide for total APOB, the concentration was 0.8574 pmol/150 µL, corresponding to 1.3998 mg/mL.

For the protein standard approach, purified human APOB100 was spiked into mouse plasma for standard addition. In the APOB100 C-terminal region, the mouse peptide FIIPGIK was baseline-separated from its human homolog FIIPGLK, enabling external calibration. The method relied on the one amino acid difference in these human and mouse peptides. APOB100 concentration determined by this approach was 0.5410 ± 0.0534 pmol/150 µL (CV% = 9.9%), in close agreement with the Lys8-FFLPDFK result. We therefore selected Lys8- FFLPDFK as the reporting peptide for APOB100, the concentration was 0.5317 pmol/150 µL, corresponding to 1.0828 mg/mL.

Based on agreement between methods, Lys8-GFEPTLEALFGK was selected as the surrogate peptide for total APOB and Lys8-FFLPDFK for APOB100. Final concentrations in the pooled mouse plasma sample then used as a standard for the total APOB ELISA were: total APOB 1.3998 mg/mL; APOB100, 1.0828 mg/mL. This plasma pool was then used in different dilutions to create a standard curve for the total APOB ELISA.

Quantitative LC–MS/MS analysis by parallel reaction monitoring (PRM). Peptide digests were analyzed on an EASY-nLC™ 1200 UHPLC system coupled to an Orbitrap Fusion Lumos Tribrid mass spectrometer (Thermo Fisher Scientific). Briefly, samples (~0.3 µg total protein equivalent) were desalted on a C18 trap column (0.1×40 mm, packed in-house with Magic C18 resin, 5 µm, 100 Å; Michrom Bioresources) at 1.5 µL/min for 8 min. Peptides were then separated on a C18 analytical column (0.075×200 mm, packed in-house with Magic C18 resin, 5 µm, 100 Å; Michrom Bioresources) at 0.3 µL/min using a multistep gradient: 1–8% solvent B (0.1% formic acid in 80% acetonitrile) in 1 min; 8–30% B in 20 min; 30–45% B in 5 min; 45–90% B in 3 min; wash at 90% B for 2 min; and re-equilibration in 1% B for 13 min. Solvent A was 0.1% formic acid in water.

The mass spectrometer was operated in positive ion PRM mode. Spray voltage was 2100 V with a 300°C ion transfer tube. Full-scan (MS1) spectra were acquired at 120,000 resolution (m/z 200), scan range m/z 300–1600, AGC target 7×10^5, and maximum injection time 50 ms. Targeted PRM scans were scheduled at retention time ±1.5 min, isolating precursor ions (charge states 1–3) with a 1.6 m/z window. MS2 spectra were acquired at 30,000 resolution (m/z 200), AGC target 5×10^4, maximum injection time 54 ms, normalized collision energy 30 eV, and scan range m/z 100–precursor upper limit. Transitions with interference were excluded, and the sum of peak areas of selected transitions were used to quantify each peptide. H:L ratios were calculated from total peak areas of Lys8-labeled (heavy) and endogenous (light) peptides.

Flow cytometry of primary hepatocytes and leukocytes. Livers were digested with liberase (#05401127001, Sigma) perfusion and percoll (#17544502, Cytva) purification, separating hepatocytes and non-parenchymal cells, the latter fraction containing the leukocytes. Flow cytometry was performed on hepatocytes and non-parenchymal cells separately. Cell lipid loading and cell viability were analyzed by BODIPY (ThermoFisher, D3922; 1 μg/1x10^6 cells) in permeabilized cells and by 7AAD (eBioscience, 00-6993), respectively. For primary mouse hepatocyte and BMDM co-culture experiments, the cells were trypsinized after stimulation and used for flow cytometry. Hepatocytes were identified as cells positive for Hoechst 33342 (see below for labeling protocol) and BMDMs were identified by cell surface CD115. Antibodies used for flow cytometry are shown in Supplemental Data 1. All flow analyses utilized directly conjugated antibodies, and nonspecific binding was blocked with anti-CD16/32 (BD Pharmingen). For analysis of intracellular staining, cells were fixed and permeabilized overnight on ice with CytoFix/CytoPerm (BD Sciences) then stained. Samples were flowed on a Symphony A3 (BD Biosciences), and analyzed by FlowJo software (Becton Dickinson). Kupffer cells were defined as $CD45^+F4/80^{Hi}CD11b^{Int}CLEC4F^+$ cells. Liver recruited monocytes were defined as $CD45^+F4/80^{Lo}CD11b^{Hi}CLEC4F^-$ cells. Each cell population was further restricted to single cells by comparing height and area side scatter pulses, and dead cells were excluded by detecting the integration of the live/dead dye. Fluorescence minus one (FMO) controls (omitting one of the targeting antibodies at a time in the presence of all other antibodies) were used for gating strategies (see **Supplemental Figure 9**).

Hepatic gene expression studies by bulk RNA sequencing (RNA-seq). For bulk RNA-seq, total RNA was isolated from frozen mouse livers using RNeasy kits (Qiagen) with on-column DNase digestion. We analyzed livers from five SLD-fed mice and ten DDC-fed mice, and eight mice per group from mice transplanted with bone marrow from WT mice, $ADAM17^{M-/-}$ mice, and $TREM2^{H-/-}$ mice. RNA (0.5 ng) was reverse transcribed into full-length amplified cDNA. Sequencing libraries were constructed using the NexteraXT DNA sample preparation kit with unique dual indexes (Illumina) to generate Illumina-compatible barcoded libraries. Libraries were pooled and quantified using a Qubit® Fluorometer (Life Technologies). Sequencing of pooled libraries was carried out on a NextSeq 2000 sequencer (Illumina) with paired-end 59-base reads, using a NextSeq™ 2000 P4 XLEAP-SBS™ sequencing kit (Illumina) with a target depth of 5

million reads per sample. Base calls were processed to FASTQs on BaseSpace (Illumina), and a base call quality-trimming step was applied to remove low-confidence base calls from the ends of reads. The FASTQs were aligned to the GRCm38 mouse reference genome, using STAR v.2.4.2a and gene counts were generated using htseq-count. QC and metrics analysis was performed using the Picard family of tools (v1.134). Differentially expressed gene (DEG) analysis was performed using DESeq2 in R. Genes were pre-filtered to include only those expressed in at least 5 samples in the DDC vs SLD comparison and at least 8 samples in the TREM2$^{H-/-}$ vs ADAM17$^{M-/-}$ comparison. P-values were adjusted for multiple testing using the Benjamini–Hochberg (BH) false discovery rate (FDR) correction. Gene Set Enrichment Analysis (GSEA) was performed on a ranked gene list based on log-fold changes using the clusterProfiler package in R. Ingenuity Pathway Analysis (IPA; Qiagen) was performed on the DEGs. Enrichment p-values were adjusted for multiple testing using the BH false discovery rate FDR correction. Only pathways with adjusted p-values <0.05 are shown.

Lipid extraction from mouse livers. The classic Folch method was used to extract lipids from frozen livers with minor modification. Liver pieces were weighed and homogenized in 0.5 mL 1M NaCl, and protein content was measured by BCA. The remaining homogenized mixture was extracted with 3.75 mL of chloroform/methanol (2:1) and H_2O was added to separate the phases. The lipid-containing organic phase at the bottom was dried under nitrogen gas and re-dissolved in isopropanol for lipid content analysis. Lipid content was normalized either by the wet weight of liver pieces or the protein content.

Real-time PCR. Gene expression in the liver and hypothalamus was measured by real-time PCR using the SYBR Green 1 detection method (ThermoScientific). Cycle threshold (Ct) values were normalized to *Rn18s* and presented as fold of control. Primer sequences are listed in Supplemental Data 1.

Primary hepatocyte and Kupffer cell isolation and hepatocyte co-culture with macrophages. Bone marrow-derived macrophages (BMDMs) of different genotypes were prepared by culturing cells flushed out of femurs and tibias in the presence of CSF-1 for 7 days. Primary hepatocytes were isolated from 6-8-week-old *Ldlr*$^{-/-}$ mice after liberase (#05401127001, Sigma) perfusion and percoll (#17544502, Cytva) purification.

Freshly isolated hepatocytes were seeded onto collagen-coated 12-well plates at 500,000 cells/well for 3 h and were labeled with Hoechst 33342 (Sigma), a cell permeable dye that labels nuclei of living cells, for 30 min to aid flow identification of hepatocytes and efferocytosed hepatocytes in the DAPI channel. Differentiated BMDM (500,000 cells/well) were either added directly onto the seated hepatocytes, or onto a trans-well insert that prevents direct contact between hepatocytes and BMDMs, and incubated with oleic acid (400 μM bound to fatty acid-free BSA at a 4:1 molar ratio) for 16-20 h. Resultant media were collected and the cells were lifted for flow analysis. Triglycerides and apolipoproteins in the media were assayed by a kit from Abcam (#ab65336) and by respective ELISAs.

While preparing primary hepatocytes, Kupffer cells in the liver leukocyte fraction of the same mice were collected and further purified by gradient centrifugation and used in flow analysis with a panel of cell-type specific markers (Supplemental Data 1).

Supplemental Table 1. Clinical characteristics of subjects with normal and elevated liver fat.

Characteristics<br>*cohort 1 – Washington U*<br>(mean ± SEM)	Normal liver fat<br>(n=10)	High liver fat<br>(n=10)	p-value<br>(Mann Whitney or t-test)
Male/Female	2/8	2/8	
Age (yr)	41.3 ± 1.8	41.3 ± 1.9	0.970
BMI (kg/m^2)	39.6 ± 1.5	39.9 ± 1.5	0.876
Fasting glucose (mg/dL)	85 ± 3	93 ± 2	**0.036**
2 h OGTT (mg/dL)	112 ± 5	161 ± 5	**8.62e-007**
Liver fat content (%)	2.9 ± 0.3	19.7 ± 3.9	**1.08e-005**
Plasma triglyceride (mg/dL)	66 ± 8	138 ± 10	**1.19e-004**
Characteristics<br>*cohort 2 – Washington U*<br>(mean ± SEM)		MASLD (n=5)	
Male/Female		2/3	
Age (yr)		55.8 ± 3.1	
BMI (kg/m^2)		34.2 ± 3.0	
Fasting glucose (mg/dL)		103 ± 6	
Plasma triglyceride (mg/dL)		131 ± 21	
Plasma cholesterol (mg/dL)		149 ± 18	
Characteristics<br>*cohort 3 – VA, Seattle*<br>(mean ± SEM)		MASLD<br>(n=12)	
Male/Female		11/1	
Age (yr)		49.8 ± 3.6	
BMI (kg/m^2)		33.1 ± 1.7	
Fasting glucose (mg/dL)		111.7 ± 8.0	
Steatosis score		1.58 ± 0.23	
Plasma TG (mg/dL)		177 ± 43	
Plasma cholesterol (mg/dL)		180 ± 17	
LDL-cholesterol (mg/dL)		111.3 ± 11.7	
HDL-cholesterol (mg/dL)		42.9 ± 6.1	

OGTT=oral glucose tolerance test

Supplemental Table 2: Plasma apolipoprotein and lipid levels in male *Ldlr*$^{-/-}$ mice transplanted with bone marrow from the four groups

Group (bone marrow genotype)	Plasma sTREM2 (ng/mL)*	Plasma APOC3 (μg/mL)	Plasma APOB100** (μg/mL)	Plasma cholesterol (mg/dL)	Plasma TG (mg/dL)
WT (*Lyz2*$^{Cre/Cre}$)	17.17 ± 1.02	1156 ± 141	5974 ± 890	629.6 ± 83.3	429.4 ± 39.6
ADAM17$^{M-/-}$ (*Adam17*$^{fl/fl}$ *Lyz2*$^{Cre/Cre}$)	11.99 ± 1.32	772 ± 97	3822 ± 316	459.6 ± 64.9	288.1 ± 44.4
TREM2$^{H-/-}$ (*Trem2*$^{-/-}$ *Lyz2*$^{Cre/Cre}$)	1.93 ± 0.23	1498 ± 243	6132 ± 711	669.5 ± 38.1	500.2 ± 38.8
ADAM17$^{M-/-}$ TREM2$^{H-/-}$ (*Adam17*$^{fl/fl}$ *Trem2*$^{-/-}$ *Lyz2*$^{Cre/Cre}$)	1.08 ± 0.15	1141 ± 119	4927 ± 320	586.1 ± 53.2	476.9 ± 61.9

Data presented as mean ± SEM (n=8,9,9,10). * Week 10, other values week 12; ** Fasting

Statistical analyses (all $p<0.05$)
sTREM2, one-way ANOVA and Tukey's multiple comparisons test:
WT vs. ADAM17$^{M-/-}$ p=6.65e-004
WT vs. TREM2$^{H-/-}$ p=3.01e-013
WT vs. ADAM17$^{M-/-}$ TREM2$^{H-/-}$ p=1.22e-013
ADAM17$^{M-/-}$ vs. TREM2$^{H-/-}$ p=3.07e-009
ADAM17$^{M-/-}$ vs. ADAM17$^{M-/-}$ TREM2$^{H-/-}$ p=2.53e-010
APOC3, one-way ANOVA and Tukey's multiple comparisons test:
ADAM17$^{M-/-}$ vs. TREM2$^{H-/-}$ p=1.46e-002
APOB100, one-way ANOVA and Tukey's multiple comparisons test:
ADAM17$^{M-/-}$ vs. TREM2$^{H-/-}$ p=3.97e-002
Plasma cholesterol, Kruskal-Wallis and Dunn's multiple comparisons test:
ADAM17$^{M-/-}$ vs. TREM2$^{H-/-}$ p=3.32e-002
Plasma TG, one-way ANOVA and Tukey's multiple comparisons test:
ADAM17$^{M-/-}$ vs. TREM2$^{H-/-}$ p=2.09e-002
ADAM17$^{M-/-}$ vs. ADAM17$^{M-/-}$ TREM2$^{H-/-}$ p=3.94e-002

Supplemental Table 3: Selected peptides used for quantifying plasma apolipoprotein Lys8 enrichment.

Proteins	Peptides and Positions	Precursor Light (m/z)	Precursor Heavy (m/z)	Charge State	RT (min)
APOB Nt	GLVHPLSTLISSSQTCQYTLDPK* [230, 252]	849.1037	851.7751	3	23.0
	GFEPTLEALFGK* [696, 707]	654.8454	658.8525	2	24.4
	INIDIPLPLGGK* [1294, 1305	625.3794	629.3865	2	23.4
APOB Ct	FFLPDFK* [3310, 3316]	457.2445	461.2516	2	23.9
	FIIPGIK* [3720, 3726]	394.2575	398.2646	2	21.5
	SLPVGNTVFDLNK* [3795, 3807]	702.3801	706.3872	2	21.0
APOC3	GYWSK* [72, 76]	640.3089	648.3231	1	15.4

A. Volcano plot of DEGs DDC vs SLD

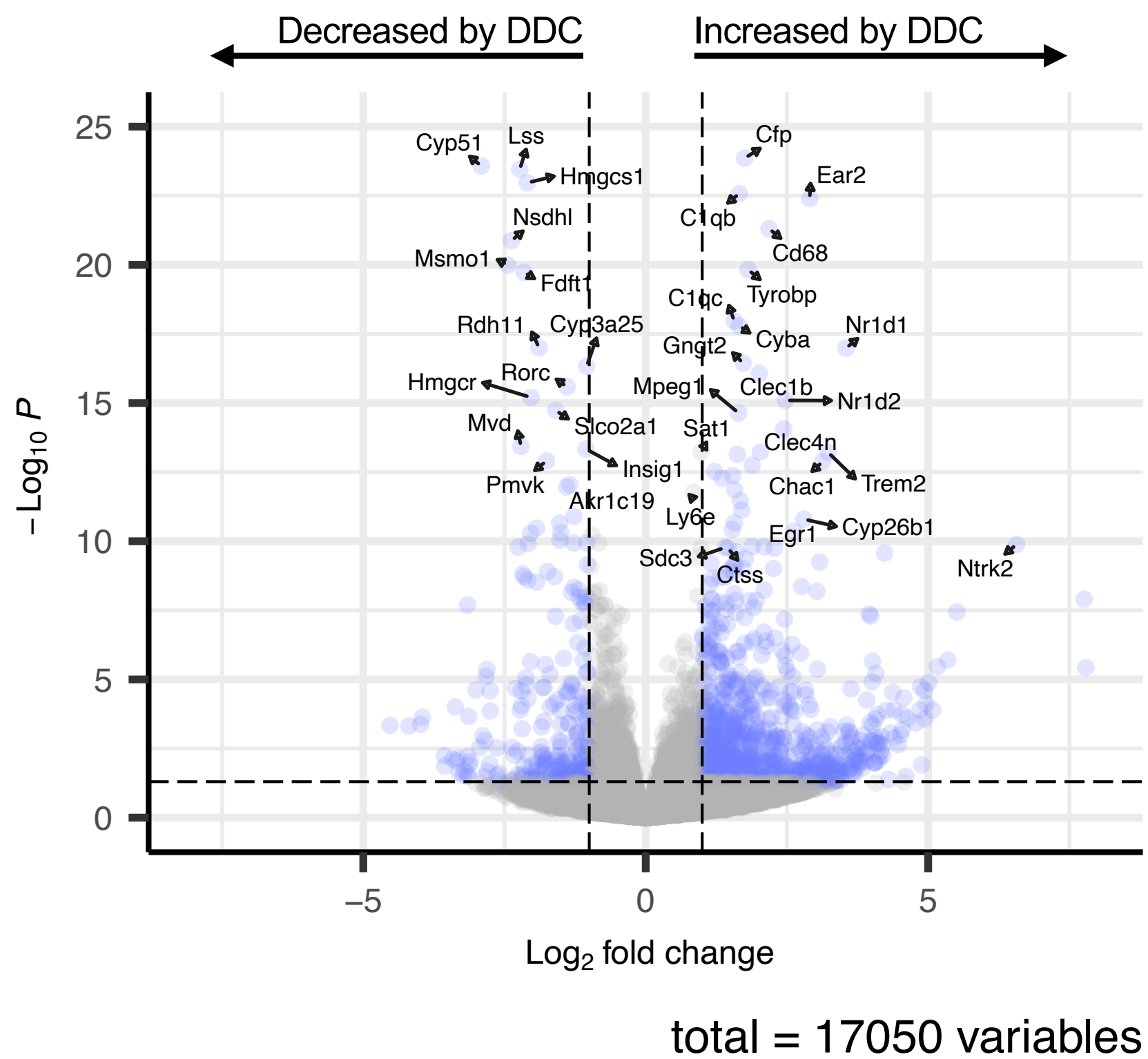

Supplemental Figure 1. Liver RNA-seq shows increased macrophage, inflammation, and ER cholesterol accumulation signatures in the mouse model of prediabetes and hepatic steatosis. Livers from the experiment shown in figure 2 were analyzed by bulk RNA-seq. (A) Volcano plot of differentially expressed genes (DEGs) in mice fed DDC versus standard laboratory diet (SLD; chow), adjusted $p<0.05$. (B) GSEA. Positive NES indicates increase in DDC-fed mice versus SLD-fed mice. See Supporting Data Values for group sizes and statistics.

B. GSEA DDC vs SLD

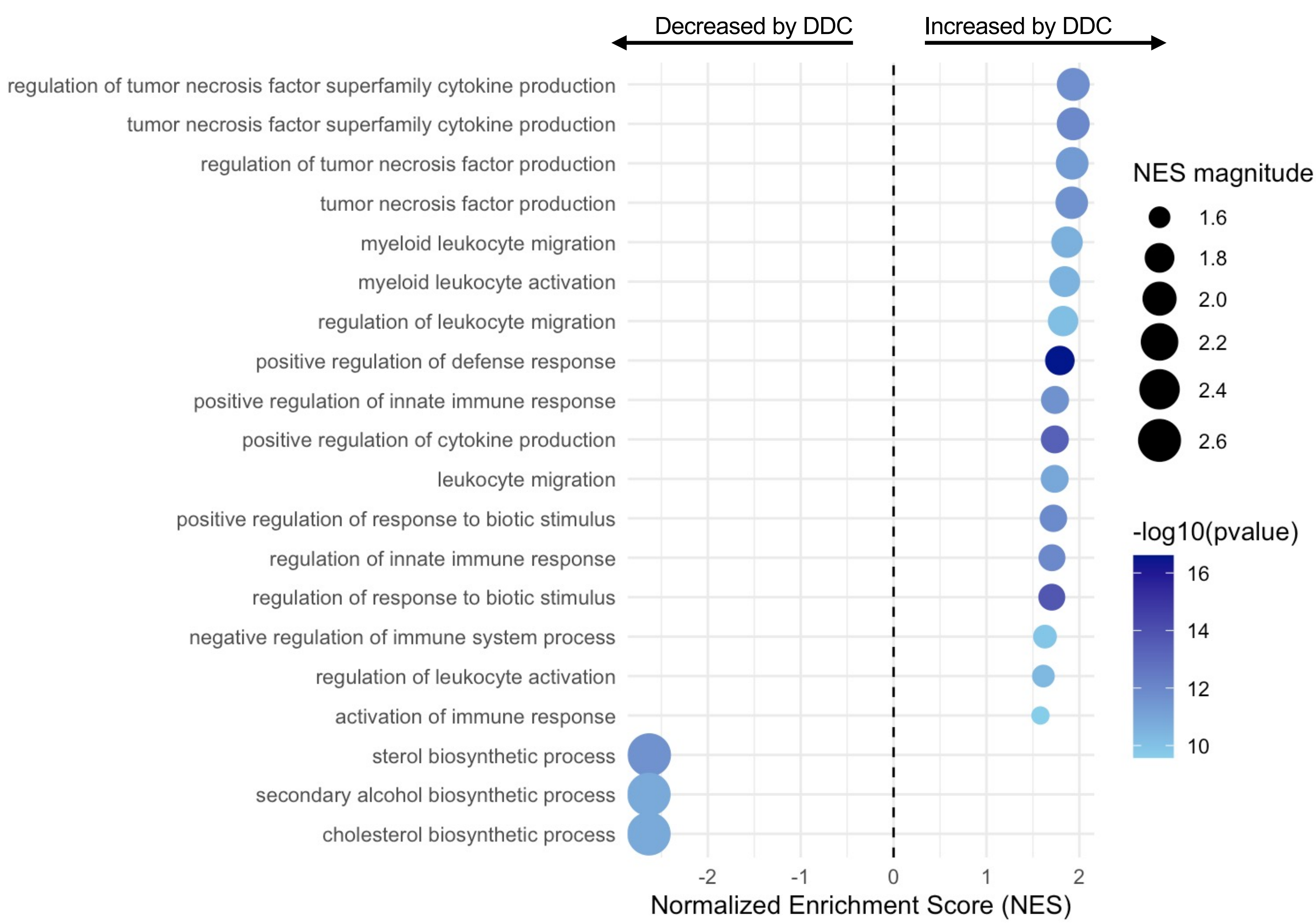

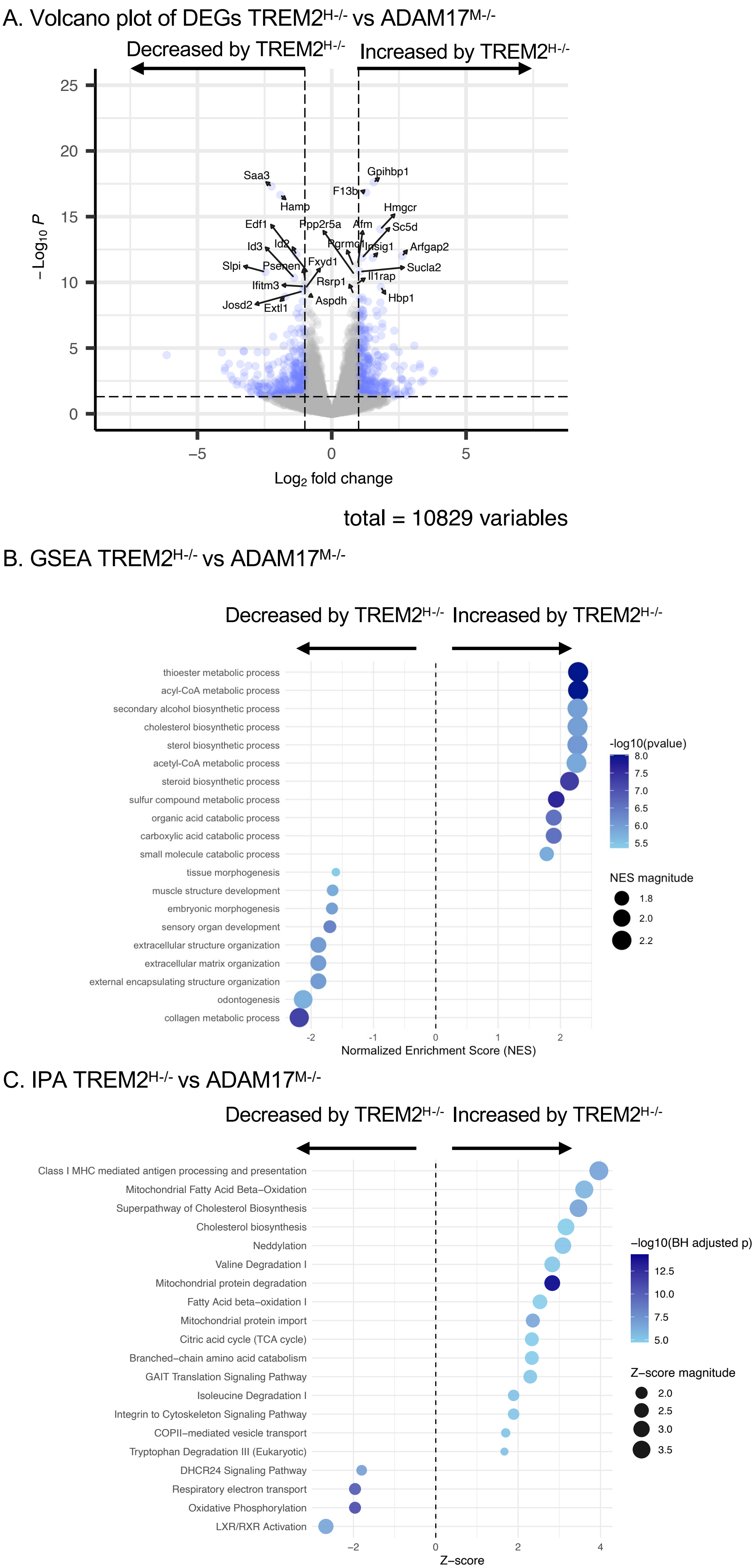

Supplemental Figure 2. Transcriptomic differences between livers from mice chimeric for hematopoietic TREM2-deficiency and myeloid cell ADAM17-deficiency. Livers from the experiment shown in figure 3 were analyzed by bulk RNA-seq. (A) Volcano plot of differentially expressed genes (DEGs) in TREM2$^{H-/-}$ vs ADAM17$^{M-/-}$ chimeras fed DDC, adjusted $p<0.05$. (B) Gene set enrichment analysis (GSEA). Positive NES indicates increases in TREM2$^{H-/-}$ mice versus ADAM17$^{M-/-}$ mice. The top 20 pathways based on adjusted p-values are shown. (C) Ingenuity pathway analysis (IPA). Positive z-scores indicate increases in TREM2$^{H-/-}$ mice versus ADAM17$^{M-/-}$ mice. The top 20 pathways based on adjusted p-values and z-scores >1.5 and <-1.5 are shown. See Supporting Data Values for group sizes and statistics.

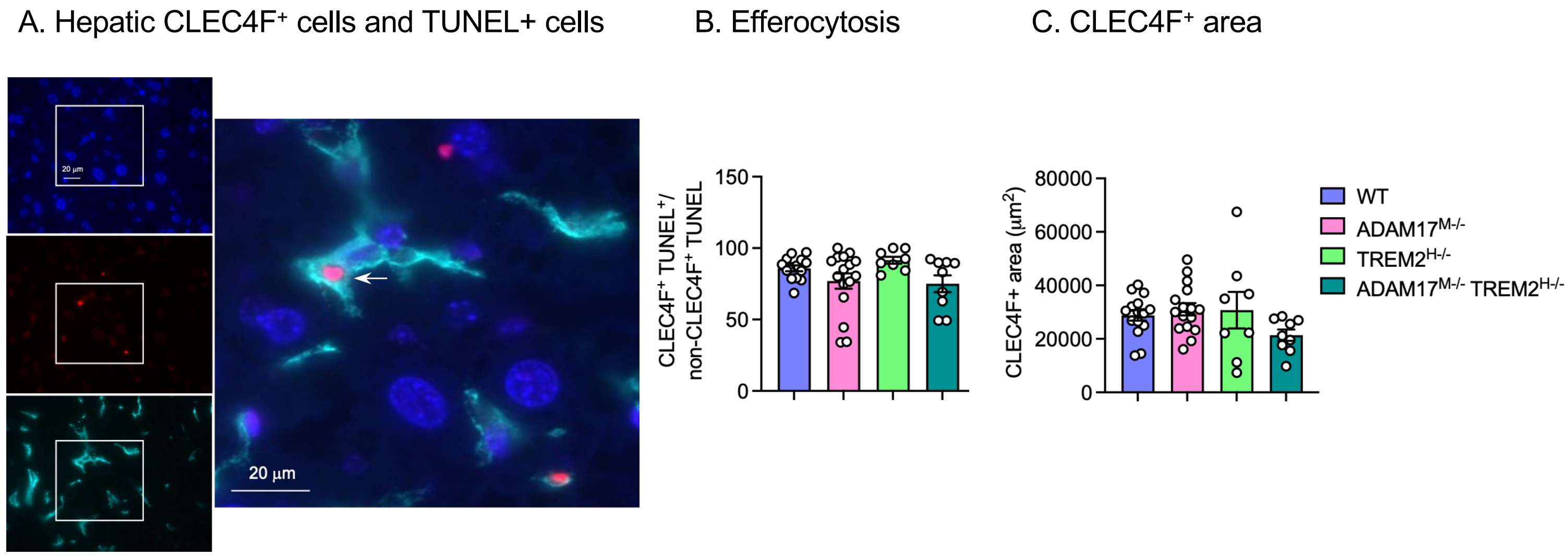

Supplemental Figure 3. Lack of effects of TREM2 on efferocytosis in the liver *in vivo*. (A-C) *Ldlr*$^{-/-}$ mice were transplanted with bone marrow from wildtype mice (WT), myeloid *Adam17*$^{-/-}$ mice, *Trem2*$^{-/-}$ mice, or myeloid *Adam17*$^{-/-}$ *Trem2*$^{-/-}$ mice. After a 7-week recovery period, the mice were fed DDC for 18 weeks. (A) Kupffer cells were quantified as CLEC4F^{+} cells (teal color) in fixed liver sections. Apoptotic cells were identified as TUNEL positive (red), and nuclei were identified by DAPI (blue). An example is indicated by the white arrow. (B) Efferocytosis was quantified as CLEC4F^{+} cells positive for TUNEL. (C) Hepatic Kupffer cell area. Mean ± SEM. Statistical analyses: one-way ANOVA with Tukey's multiple comparisons test (B-C). See Supporting Data Values for group sizes and statistics.

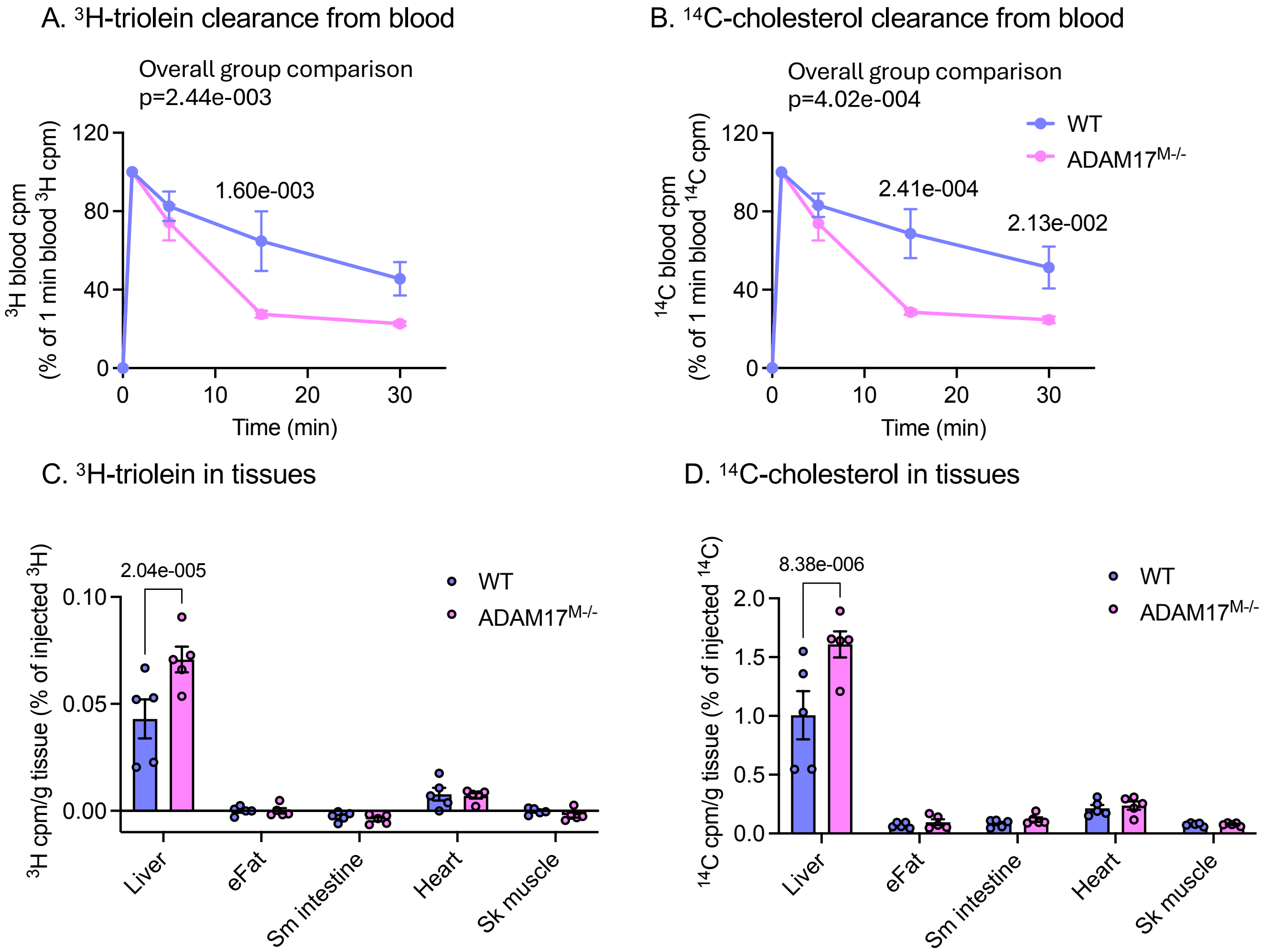

Supplemental Figure 4. Myeloid cell-targeted ADAM17-deficiency increases TRL triglyceride and cholesterol clearance rates. Lyz2$^{fl/fl}$ mice (wildtype; WT) and myeloid *Adam17*$^{-/-}$ mice were fed DDC for 13 weeks and treated weekly with a liver-targeted Ldlr GalNAc ASO administered i.p. Clearance of radiolabeled chylomicrons was measured in mice fasted for 4 h before injection of radiolabeled chylomicron via the retro-orbital sinus. Blood was collected at the indicated time-points, and radioactivity was measured by scintillation counter. At the end of the experiment (30 min after injection), liver, epidydimal fat, small intestine, heart, and skeletal muscle were collected, and radioactivity was measured by scintillation counter. Radiolabeled chylomicrons were generated from fasted *Gpihbp1*$^{-/-}$ mice orally gavaged with olive oil mixed with [9,10-^{3}H(N)]-Triolein (Perkin Elmer, NET431001MC) and [4-^{14}C]-Cholesterol (Perkin Elmer, NEC018050UC). Mean ± SEM. Statistical analyses: two-way ANOVA with Šídák's multiple comparisons test (A-D). See Supporting Data Values for group sizes and statistics.

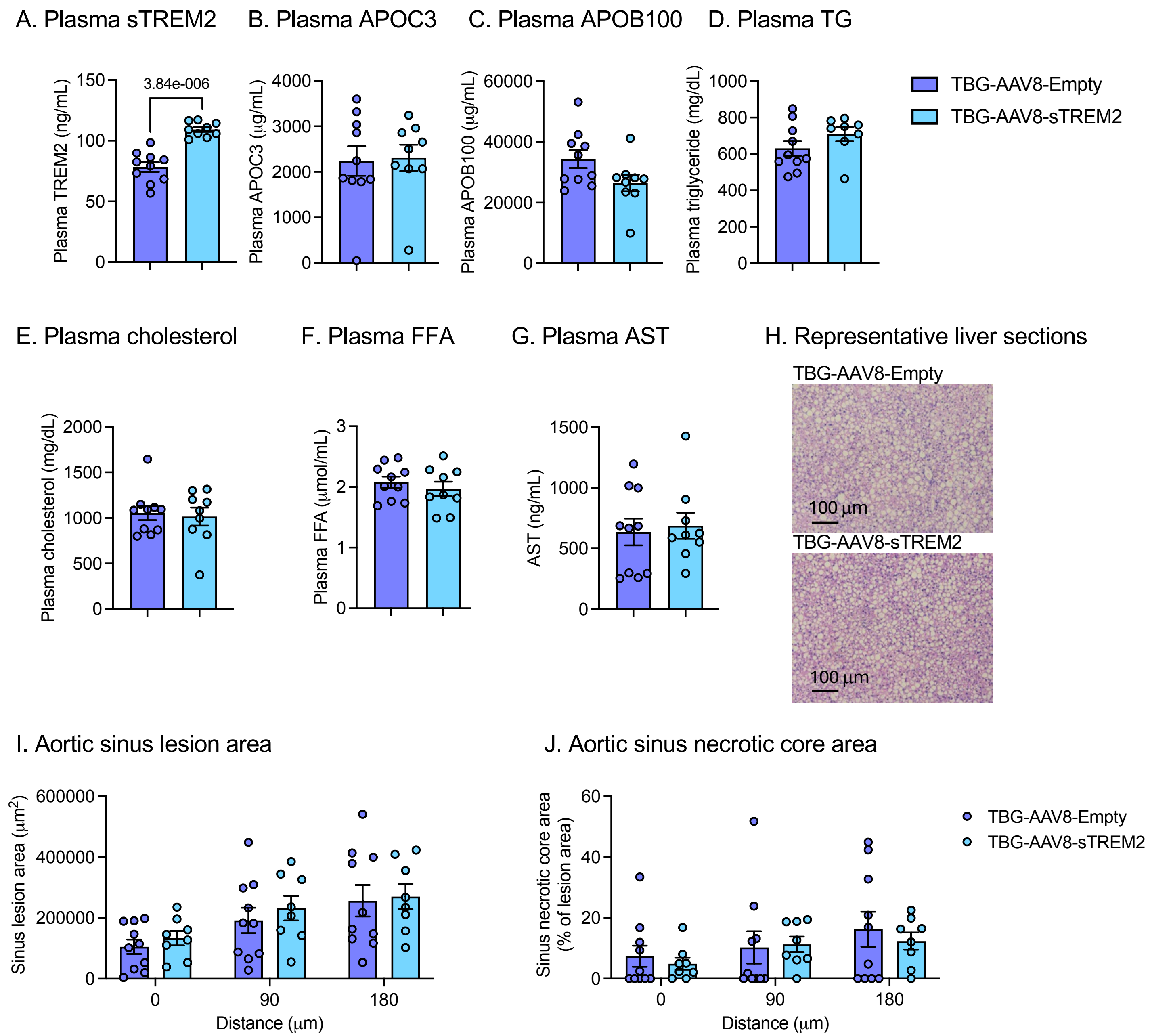

Supplemental Figure 5. Elevated sTREM2 does not increase plasma APOC3, APOB100, or atherosclerosis. *Ldlr*$^{-/-}$ male mice were injected with a liver-targeted TBG-AAV8 expressing the soluble fragment of TREMs (TBG-AAV8-sTREM2) or empty control (TBG-AAV8-Empty). The mice were then fed DDC for 18 weeks. At the end of the study, plasma sTREM2 levels were measured by ELISA (A) to confirm elevated levels. (B-C) Plasma APOC3 and APOB100 levels at study end. (D-F) Plasma triglyceride (TG), cholesterol and free fatty acid (FFA) levels at the end of the study. (G) Plasma AST levels at study end. (H) H&E-stained liver sections showing similar steatosis in the control-TBG-AAV8- and sTREM2-TBG-AAV8-injected mice. (I-J) The aortic sinus was serial sectioned, and the sections were stained using a Movat's pentachrome stain. Lesion area (I) and lesion necrotic core area (J) were analyzed at the indicated distances from the aorta. Mean ± SEM. Statistical analyses: unpaired t test (A, C, F), Mann Whitney (B, D-E, G), two-way ANOVA with Šídák's multiple comparisons test (I-J). See Supporting Data Values for group sizes and statistics.

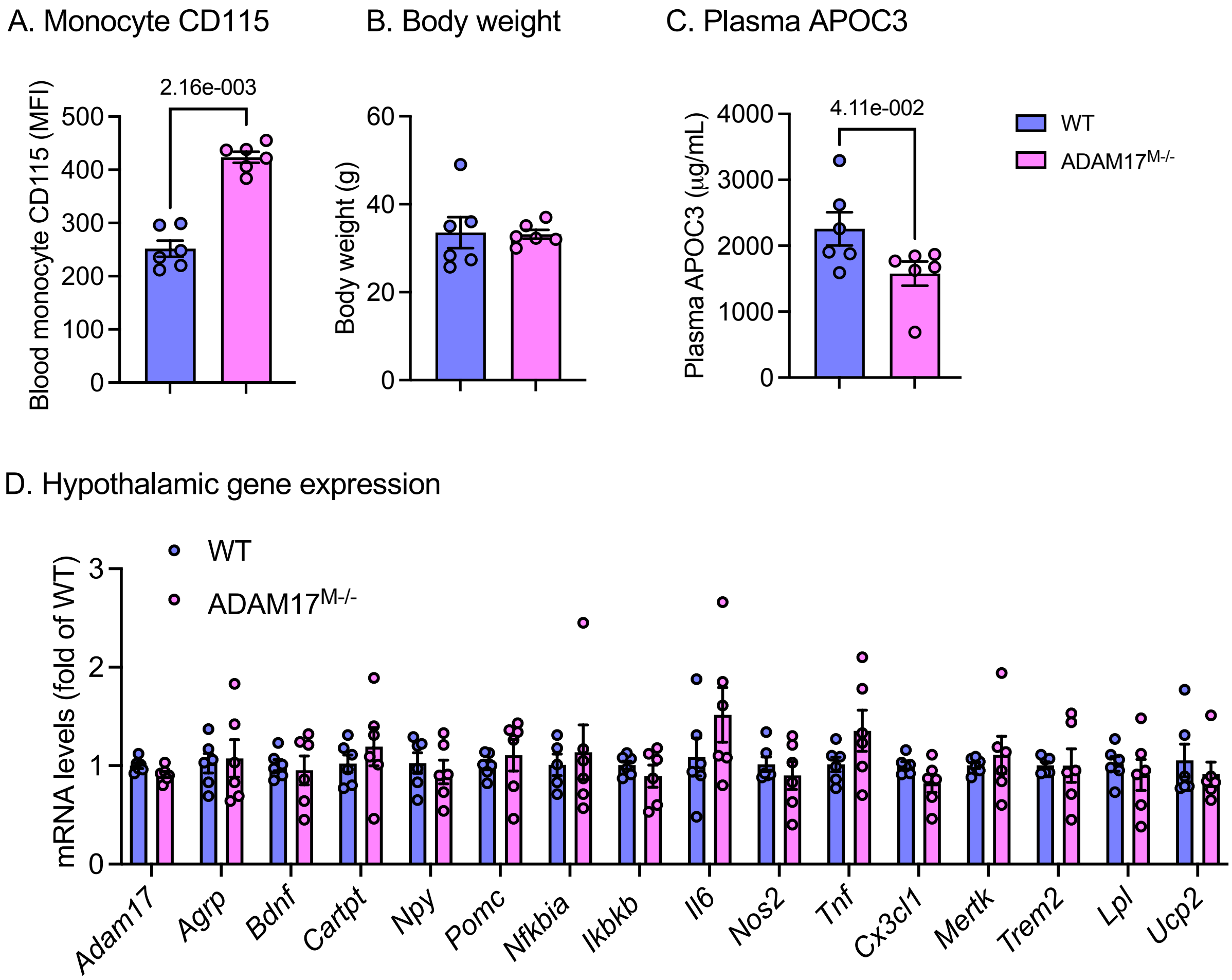

Supplemental Figure 6. Myeloid cell-targeted ADAM17-deficiency does not alter hypothalamic gene expression. *Ldlr*$^{-/-}$ mice were transplanted with bone marrow from wildtype mice (WT) or myeloid *Adam17*$^{-/-}$ mice. After a 7-week recovery period, the mice were fed DDC for 18 weeks. (A). Blood monocyte surface CD115 levels confirming that ADAM17 deletion results in the predicted lack of CD115 shedding. (B). Body weights at the end of the experiment. (C). Plasma APOC3 levels at the end of the experiment. (D). Hypothalamic mRNA levels were measured by real-time PCR. Mean ± SEM. Statistical analyses: Mann Whitney (A-C), two-way ANOVA with Šídák's multiple comparisons test (D). See Supporting Data Values for group sizes and statistics.

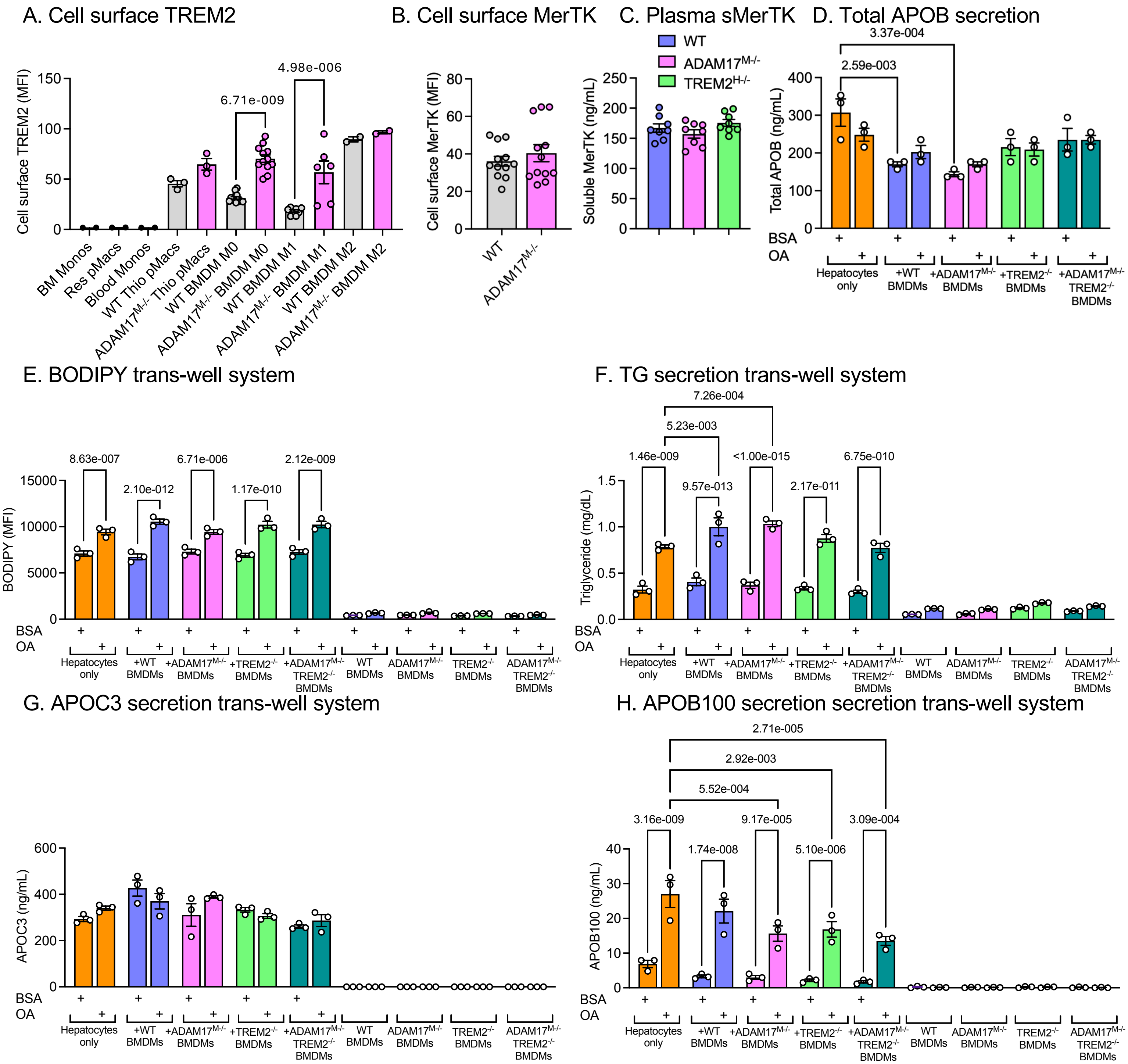

Supplemental Figure 7. Macrophage TREM2's effects on TG and APOB100 secretion require physical contact with hepatocytes. (A) Cell surface TREM2 levels in macrophage populations from wildtype or ADAM17-deficient mice. M0 BMDM are unstimulated post 7-day CSF-1 cultured bone marrow cells; M1 is M0 stimulated with LPS (100 ng/mL) for 24 h; M2 is M0 stimulated with IL-4 (20 ng/mL) for 24 h. (B) Cell surface MerTK in M0 BMDMs. (C) Plasma sMerTK in the groups of mice in figure 3. (D) Hepatocytes co-cultured for 20 h with and without BMDMs from wildtype (WT), myeloid-targeted ADAM17$^{M-/-}$ mice, *Trem2*$^{-/-}$ mice, and mice deficient in both TREM2 and myeloid ADAM17 in the presence of oleic acid (OA) bound to BSA, or with BSA alone. Total APOB levels in the conditioned media were measured by ELISA. (E-H). Hepatocytes and BMDMs were separated physically by a trans-well system. (E) Lipid loading of hepatocytes and BMDMs measured as BODIPY (MFI) by flow cytometry. (F) TGs secreted into the conditioned media. (G) APOC3 secretion into the conditioned media. (H) APOB100 secretion into the conditioned media. Mean ± SEM. Statistical analyses: two-way ANOVA with Šídák's multiple comparisons test (A), unpaired t test (B), one-way ANOVA with Tukey's multiple comparisons test (C), two-way ANOVA with Tukey's multiple comparisons test (D-H). See Supporting Data Values for group sizes and statistics.

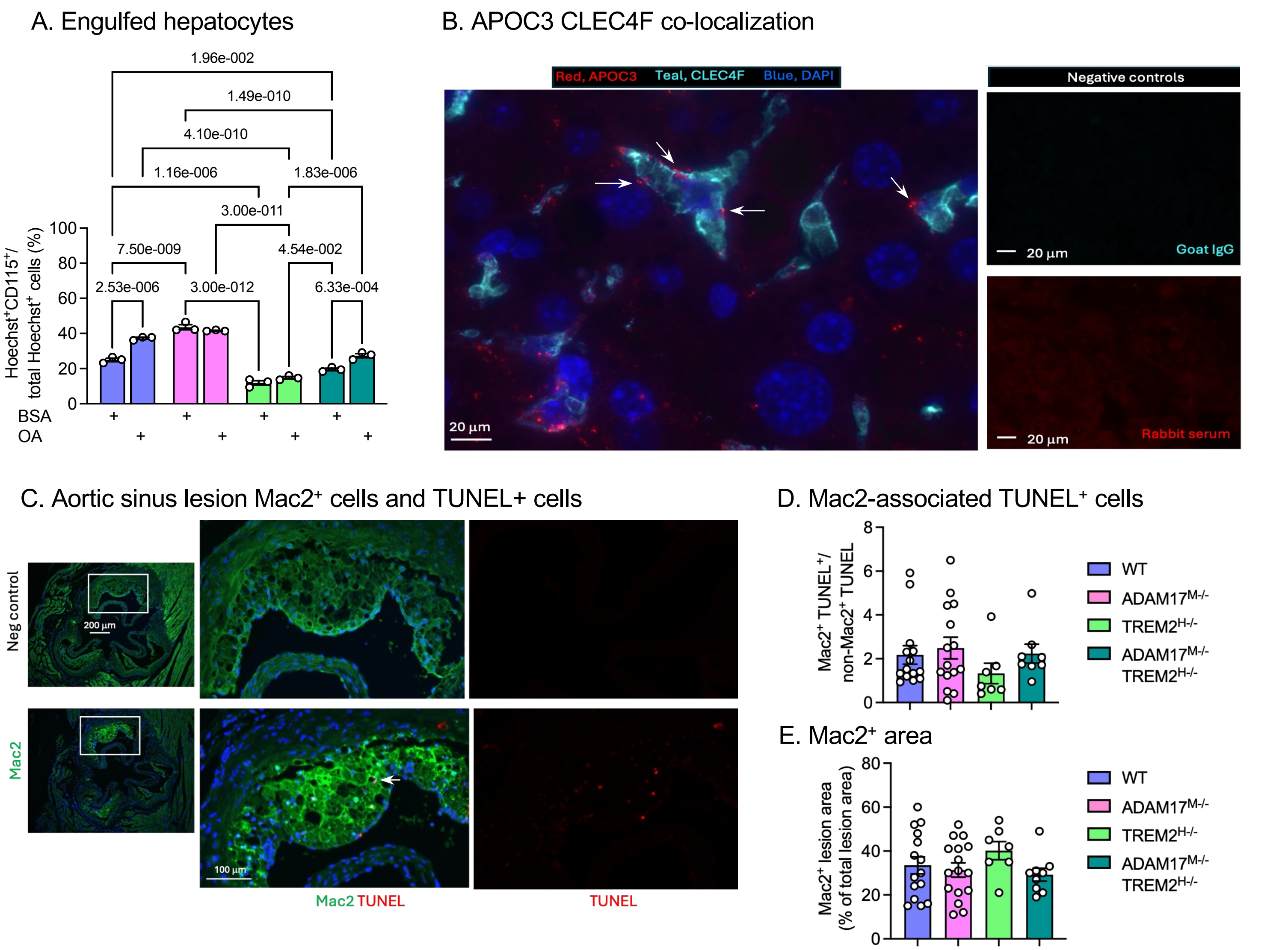

Supplemental Figure 8. Lack of effects of TREM2 on efferocytosis *in vivo*. (A) Hepatocytes were isolated from *Ldlr*$^{-/-}$ mice labeled with Hoechst 33342 and immediately co-cultured for 20 h with and without BMDMs from the four groups of mice in the presence of oleic acid (OA) bound to BSA or with BSA alone. Engulfed hepatocytes were quantified by flow cytometry (Hoechst^{+} macrophages/total hepatocytes). (B) APOC3 (red, white arrows) CLEC4F (teal) co-localization in a liver from an ADAM17$^{M-/-}$ mouse. APOC3 immunoreactivity could indicate APOC3 bound to lipoproteins, lipids, receptors, or free APOC3. (C-E) *Ldlr*$^{-/-}$ mice were transplanted with bone marrow from wildtype mice (WT), myeloid *Adam17*$^{-/-}$ mice, *Trem2*$^{-/-}$ mice, or myeloid *Adam17*$^{-/-}$ *Trem2*$^{-/-}$ mice. After a 7-week recovery period, the mice were fed DDC for 18 weeks. (C-E) Mac2^{+} TUNEL^{+} cells in the aortic sinus were quantified in the same groups of mice. Mac2^{+} cells (green); TUNEL^{+} cells (red). Statistical analyses: two-way ANOVA with Tukey's multiple comparisons test (A), Kruskal-Wallis with Dunn's multiple comparisons test (D-E). See Supporting Data Values for group sizes and statistics.

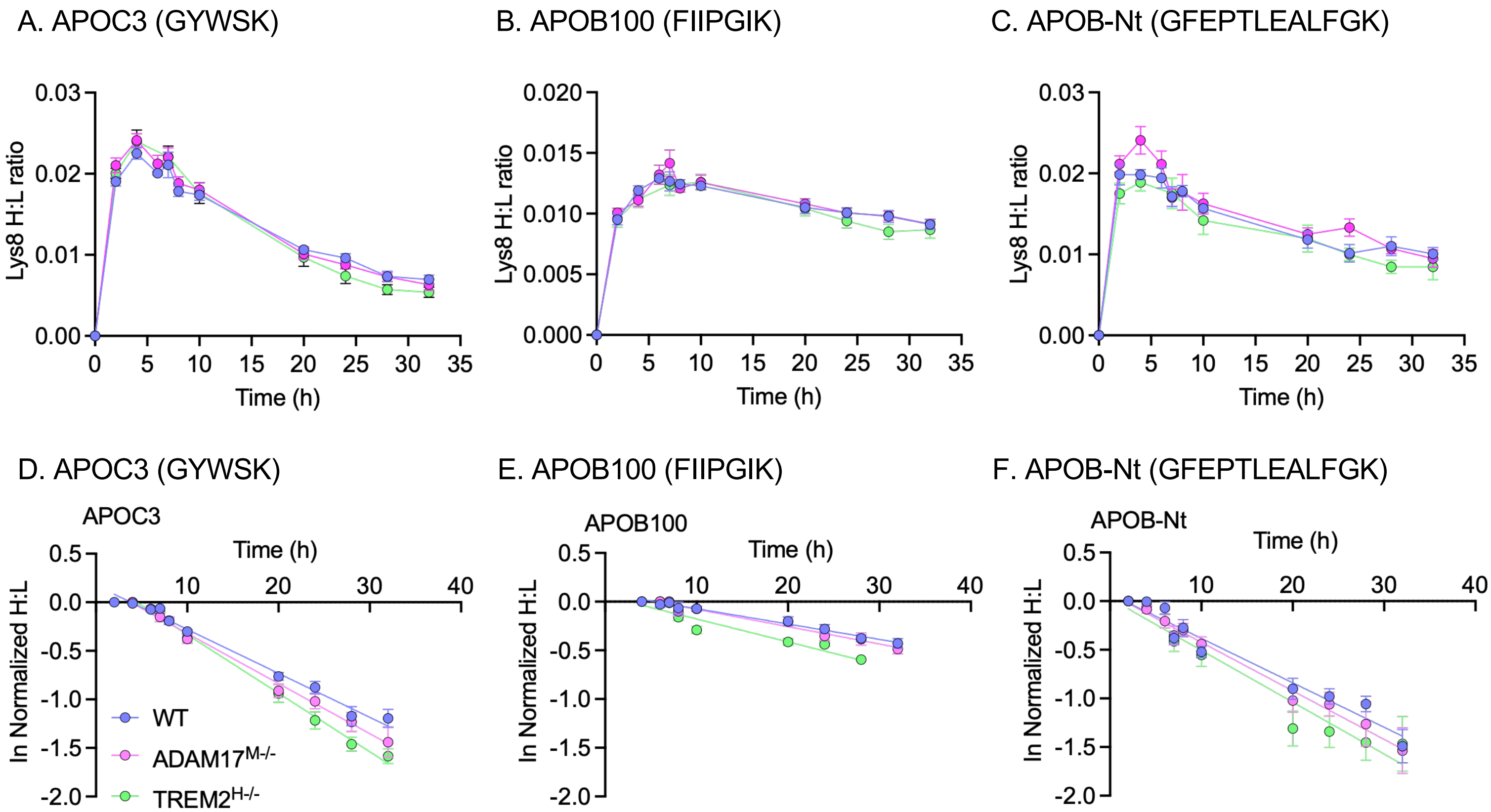

Supplemental Figure 9. APOC3 and APOB enrichment curves and monoexponential slope analysis. APOC3 and APOB enrichment curves and monoexponential slope analysis used to calculate kinetics in Figure 3L-M.

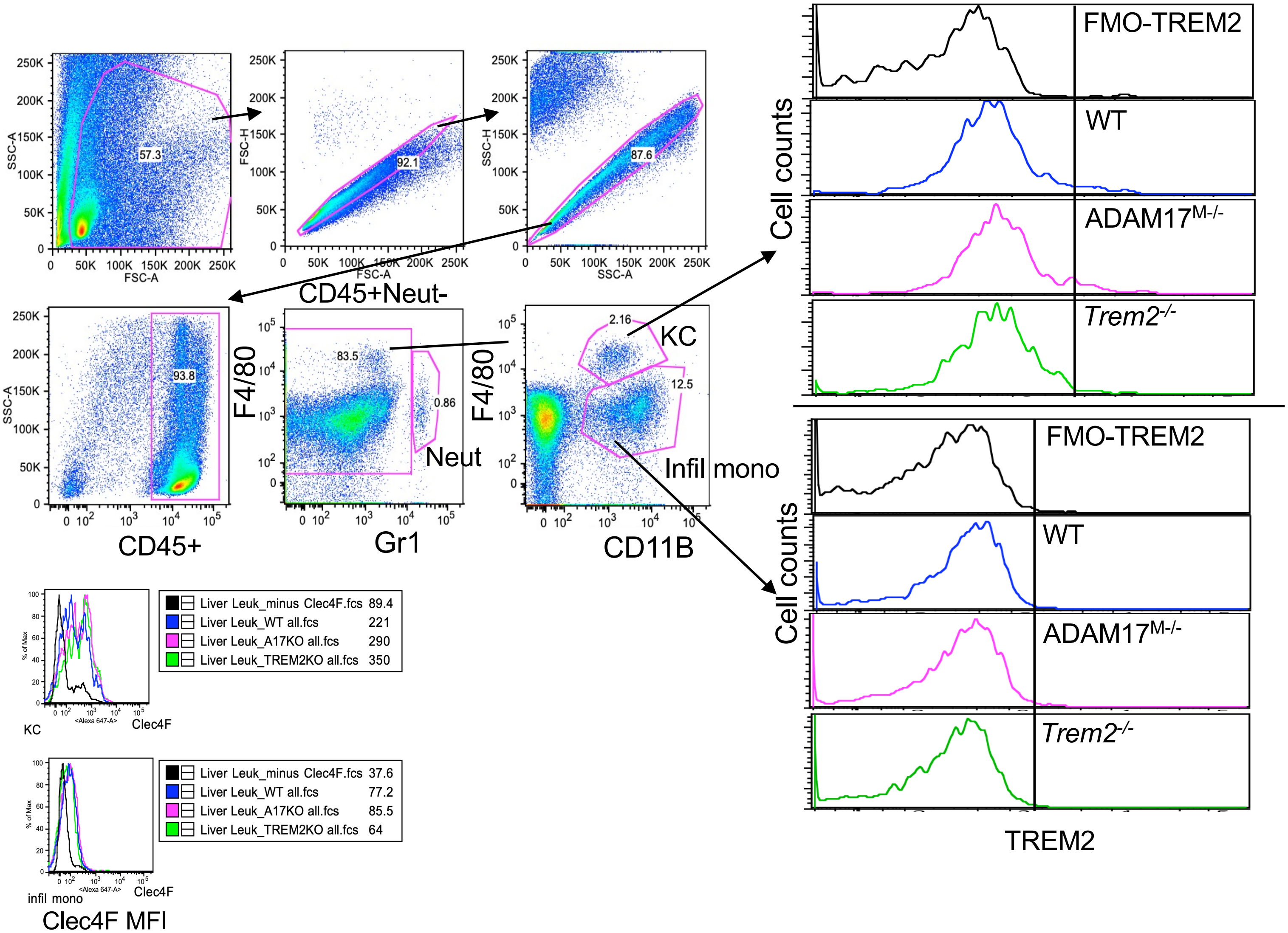

Supplemental Figure 10. Liver leukocyte flow gating strategy. Livers from age-matched SLD-fed wildtype (WT; C57BL/6J) mice, ADAM17$^{M-/-}$ mice, and *Trem2*$^{-/-}$ mice were dissected and digested (see Methods). The mice had not undergone bone marrow transplants. The leukocyte fraction was stained with specific antibodies, as shown above. Liver macrophage populations and neutrophils were identified by flow cytometry, each compared to its own FMO on a Symphony A3 flow cytometer. FMO, fluorescence minus one; Infil mono, infiltrated monocytes/macrophages; KC, Kupffer cells
