# Supplementary material for: Macrophage TREM2 is a triglyceride-rich lipoprotein receptor that undergoes shedding in prediabetic hepatic steatosis
Source: JCI Insight. 2026 Aug 27;11(19):e202675. doi: 10.1172/jci.insight.202675 (PMC13726429; doi:10.1172/jci.insight.202675)
Supplement: Supplementary file 2 [file jciinsight-11-202675-s120.pdf]

Major Resources Document

Mouse Models

Strain	Vendor or Source	Background Strain	Sex	Other Information (e.g. breeding scheme for F1, F2…if applicable)	Persistent ID / URL
B6.129S7-Ldlrtm1Her/J	JAX (Strain #000664)	C57BL/6J	M	This strain was purchased for mouse breeding to generate all experimental mice	https://www.jax.org/strain/002207
C57BL/6J-Trem2em2Adiuj/J	JAX (Strain# 027197	C57BL/6J	F, M	This strain was purchased for mouse breeding to generate bone marrow donors for transplantation	https://www.jax.org/strain/027197#
B6.129P2-Lyz2tm1(cre)Ifo/J	JAX (Strain #004781)	C57BL/6J	F, M	This strain was purchased for mouse breeding to generate controls ($Lyz2^{Cre/Cre}$), myeloid-targeted ADAM17-deficient mice ($ADAM17^{M-/-}$), *$Trem2^{-/-}$* $Lyz2^{Cre/Cre}$ mice, and $ADAM17^{M-/-}$ *$Trem2^{-/-}$* $Lyz2^{Cre/Cre}$ mice	https://www.jax.org/strain/004781
Adam17fl/fl Lyz2Cre/Cre	In house	C57BL/6J	M, F	$Adam17^{fl/fl}$ mice (generation described in PMC3622831) were generated in-house and crossed with $Lyz2^{Cre/Cre}$ mice	

Antibodies for flow cytometry

Target antigen	Vendor or Source	Catalog #	Working concentration	Lot # (preferred but not required)	Persistent ID / URL
CD45-PE/Dazzle 594	Biolegend	30-F11	1:500 (0.1 µg/100 µL)		
CD115-APC	eBioscience	AFS98	1:100 (0.2 µg/100 µL)		
GR1-AF700	Invitrogen	RB6-8C5	1:1000 (0.05 µg/100 µL)		
TREM2-PE	R&D	FAB17291P	5 µL/test		
CD11B-PECy7	eBioscience	M1/70	1:500 (0.1 µg/100 µL)		
F4/80-BV605	Biolegend	BM8	1:200 (0.2 µg/100 µL)		
Viability dye BV421	Biolegend	423114	1 uL/test		
Ly6G-BUV737	Invitrogen	1A8-Ly6g	1:200 (0.2 µg/100 µL)		
Clec4F-AF647	Biolegend	3E3F9	1:200 (0.5 µg/100 µL)		
CD3-BB700	Invitrogen	145-2C11	1:200 (0.2 µg/100 µL)		
Bodipy 493/503	Invitrogen	D3922	1:200 (0.1 µg/100 µL)		
MerTK-PE	eBioScience	12-5751-82	1:200 (1µg/test)		

Antibodies and reagents for immunohistochemical staining

Target antigen	Vendor or Source	Catalog #	Working concentration	Lot # (preferred but not required)	Persistent ID / URL
CLEC4F (goat IgG)	R&D	AF2784	4 µg/mL		
AF647 donkey anti goat IgG	InVitrogen	A21447	1:500		
DAPI fluoromount-G	Southern Biotech	0100-20	mounting		
APOB (goat IgG)	Abcam	Ab7616	1:500		
APOC3 (Rabbit serum)	Ionis PharmaceuticalsCarlsbad, CA	gift	1:1000		

ADRP/PLIN2 (Rabbit IgG)	ProteinTech	15294-1-AP	1:300		
AF555 goat anti rabbit IgG	InVitrogen	A21428	1:500		
Hoechst 33342	Thermo	62249	1:1000		

Primer sequences (mouse)

Gene	Forward primer (5'-3')	Reverse primer (5'-3')
Rn18s	CATTAAATCAGTTATGGTTCCTTTGG	CCCGTCGGCATGTATTAGCT
Apoc3	TACAGGGCTACATGGAACAAGC	CAGGGATCTGAAGTGATTGTCC
Adam17	GGCTCATAGACAGAGAGCCAT	CGATGATCTGCTACCACCAGTA
Agrp	AGGGCATCAGAAGGCCTGACCAGG	CATTGAAGAAGCGGCAGTAGCACGT
Bdnf	CCATAAGGACGCGGACTTGTAC	GAGGAGGCTCCAAAGGCACTT
Cartpt	CTCCTGGGCGCCGCCCTGCTGC	CATGGGGACTTGGCCGTACTTC
Npy	ACCAGGCAGAGATATGGCAAGA	GGACATTTTCTGTGCTTTCTCTCATTA
Pomc	CGCTCCTACTCCATGGAGCACTT	TCGCCTTCCAGCTCCCTCTTG
Nfkbia	TGCCTGGCCAGTGTAGCAGTCTT	CAAAGTCACCAAGTGCTCCACGAT
Ikbkb	CTGCGGGAAGGAGCTGTC	GGCTTCAGGTCTCGATGGAT
Il6	GTGGCTAAGGACCAAGACCA	GGTTTGCCGAGTAGACCTCA
Nos2	CCTGGTACGGGCATTGCT	GCTCATGCGGCCTCCTT
Tnf	CATCTTCTCAAAACTCGAGTGACAA	TGGGAGTAGATAAGGTACAGCCC
Cx3cl1	CGCGTTCTTCCATTTGTGTA	CTGTGTCGTCTCCAGGACAA
Mertk	ACGTTGGTGGATACGTGCAT	CTCTTCCCACTTCTCGGCAG
Trem2	CAGTGTCAGAGTCTCCGAGG	ACCCAGAGATCTCCAGCATC
Lpl	CCATCCGTGTGATTGCAGAG	CCTTGCTGGGGTTTTCTTCA
Ucp2	CCGGGGCCTCTGGAAAG	CCCAAGCGGAGAAAGGA

Cultured Cells

Name	Vendor or Source	Sex (F, M, or unknown)	Persistent ID / URL
Primary mouse bone marrow-derived macrophages	Experimental mice used in this study	F, M	
Primary mouse hepatocytes	Experimental mice used in this study	F, M	
Primary mouse thioglycolate-elicited macrophages	Experimental mice used in this study	F, M	

Data & Code Availability

Description	Source / Repository	Persistent ID / URL
Original data are available in the Original Data Supplement		
Liver bulk RNA-seq	NCBI GEO	GSE343509
Liver bulk RNA-seq	NCBI GEO	GSE343510

ARRIVE GUIDELINES

The ARRIVE guidelines (https://arriveguidelines.org/) are a checklist of recommendations to improve the reporting of research involving animals. Key elements of the study design should be included below to better enable readers to scrutinize the research adequately, evaluate its methodological rigor, and reproduce the methods or findings.

Study Design

Groups	Sex	Age	Number (prior to experiment)	Number (after termination)	Littermates (Yes/No)	Other description
Figure 2	M	8-12 w	15	15	yes	
Figure 3	M	8-12 w	27	26	yes	1 dead due to transplant failure
Figure 4	M	8-12 w	26	26	yes	
Figure 5	M,F	8-12 w	12	12	yes	BMDM-derived for Figure 5A-5D<br>5E and 5F are the same cohort as Figure 6
Figure 6	M	8-12 w	53	52	yes	Same 2 cohorts as in Figure 3 and Figure 4G-I
Suppl. Fig. 1	M	8-12 w	15	15	yes	Same cohort as in Figure 2, but RNA-seq was limited to n=8/group
Suppl. Fig. 2	M	8-12 w	27	26	yes	Same cohort as in Figure 3, but RNA-seq was limited to n=8/group
Suppl. Fig. 3	M	8-12 w	53	52	yes	Same 2 cohorts as in Figure 6
Suppl. Fig. 4	M	8-12 w	10	10	yes	
Suppl. Fig. 5	M	8-12 w	19	19	yes	1 mouse in the TREM2-AAV group euthanized due to dermatitis
Suppl. Fig. 6	M	8-12 w	12	12	yes	
Suppl. Fig. 7	M	8-12 w	42	42	yes	Data in Fig. S7D same cohort as in Figure 4B-F
Suppl. Fig. 8	M	8-12 w	53	52	yes	Same cohorts as in Figure 4B-F and Figure 6
Suppl. Fig. 9	M	8-12 w	27	26	yes	Same cohort as Figure 3L-M
Suppl. Fig.10	M	8-12 w	8	8	no	

Sample Size:

Group sizes for in vivo experiments were determined by power calculations prior to the study.

Inclusion Criteria

Male mice 8-12 weeks of age.

For BMDM cultures, either male or female (matched for any giving experiment) bone marrows were used.

Exclusion Criteria

Differences in the number of mice/treatment group are due to the use of littermate controls from our colonies and to mice excluded by the following pre-determined criteria: malocclusion or dermatitis.

Randomization

Littermate mice were randomized into different groups based on similar body weights.

Blinding

All analyses were done in a masked fashion. Blinding was performed by coding the samples.
